# Impaired renal reserve contributes to preeclampsia via the kynurenine and soluble fms–like tyrosine kinase 1 pathway

**DOI:** 10.1172/JCI158346

**Published:** 2022-10-17

**Authors:** Vincent Dupont, Anders H. Berg, Michifumi Yamashita, Chengqun Huang, Ambart E. Covarrubias, Shafat Ali, Aleksandr Stotland, Jennifer E. Van Eyk, Belinda Jim, Ravi Thadhani, S. Ananth Karumanchi

**Affiliations:** 1 Department of Medicine, Cedars-Sinai Medical Center, Los Angeles, California, USA.; 2 EA-3801, Université de Reims Champagne-Ardenne, Reims, France.; 3 Department of Pathology and Laboratory Medicine, and; 4 Department of Biomedical Sciences and Smidt Heart Institute, Cedars-Sinai Medical Center, Los Angeles, California, USA.; 5 Department of Medicine, Jacobi Medical Center, Albert Einstein College of Medicine, Bronx, New York, USA.; 6 Department of Medicine, Massachusetts General Hospital, Harvard Medical School, Boston, Massachusetts, USA.

**Keywords:** Nephrology, Reproductive Biology, Hypertension, Obstetrics/gynecology

## Abstract

To understand how kidney donation leads to an increased risk of preeclampsia, we studied pregnant outbred mice with prior uninephrectomy and compared them with sham-operated littermates carrying both kidneys. During pregnancy, uninephrectomized (UNx) mice failed to achieve a physiological increase in the glomerular filtration rate and during late gestation developed hypertension, albuminuria, glomerular endothelial damage, and excess placental production of soluble fms–like tyrosine kinase 1 (sFLT1), an antiangiogenic protein implicated in the pathogenesis of preeclampsia. Maternal hypertension in UNx mice was associated with low plasma volumes, an increased rate of fetal resorption, impaired spiral artery remodeling, and placental ischemia. To evaluate potential mechanisms, we studied plasma metabolite changes using mass spectrometry and noted that l-kynurenine, a metabolite of l-tryptophan, was upregulated approximately 3-fold during pregnancy when compared with prepregnant concentrations in the same animals, consistent with prior reports suggesting a protective role for l-kynurenine in placental health. However, UNx mice failed to show upregulation of l-kynurenine during pregnancy; furthermore, when UNx mice were fed l-kynurenine in drinking water throughout pregnancy, their preeclampsia-like state was rescued, including a reversal of placental ischemia and normalization of sFLT1 levels. In aggregate, we provide a mechanistic basis for how impaired renal reserve and the resulting failure to upregulate l-kynurenine during pregnancy can lead to impaired placentation, placental hypoperfusion, an antiangiogenic state, and subsequent preeclampsia.

## Graphical abstract

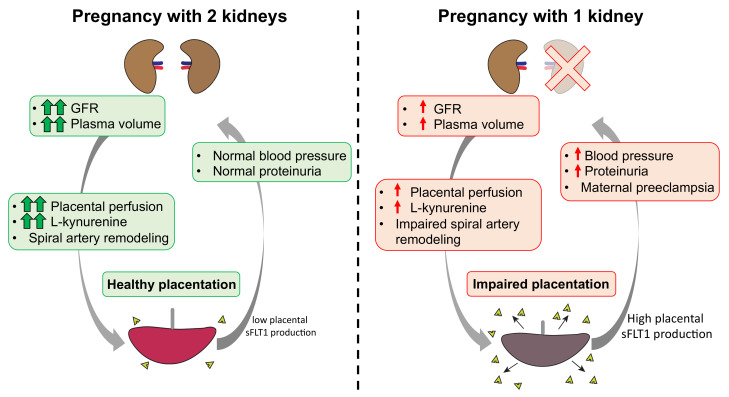

## Introduction

Pregnancy is an adaptive state of net sodium and volume expansion for the purpose of increasing perfusion of the placenta and fetus. Blood flow to the uterus can increase 5-fold to keep up with the metabolic demands of the growing fetus and placenta. The kidney is a central player in the expansion of plasma volume in normal pregnancy (1). During pregnancy, the glomerular filtration rate (GFR) increases by 50%, and renal plasma flow increases by 80% compared with nonpregnant (NP) levels. These changes are associated with reductions in serum creatinine, urea, and uric acid levels (1). In women with reduced renal reserve, such as that seen in women with a prior history of acute kidney injury (2, 3), in renal allograft recipients (4), or in women with chronic kidney disease (5), a lack of increase in the GFR during pregnancy is thought to lead to an increased risk of preeclampsia, although the precise mechanisms are largely unknown.

The study of pregnancy outcomes in women with 1 kidney represents a natural experiment to evaluate the consequences of impaired renal reserve. Multiple epidemiological studies have recently reported that live kidney donors are at increased risk for developing preeclampsia (6–8). If true, the mechanistic basis for this risk is unknown, and, indeed, this observation may simply reflect hyperfiltration by the remaining kidney, leading to mild proteinuria — which may be misclassified as preeclampsia. Prior animal studies provided conflicting data on pregnancy phenotypes in animals with impaired renal reserve (9, 10). Here, to mimic women who are kidney donors, we characterized pregnancy phenotypes in uninephrectomized (UNx) mice and report that the loss of 1 kidney led to decreased placental perfusion and impairment of spiral artery (SA) remodeling at the maternal-fetal interface. Impaired placental perfusion and accompanying placental ischemia led to a maternal preeclampsia–like phenotype. A maternal preeclampsia–like state in UNx mice was associated with placental ischemia, upregulation of soluble fms–like tyrosine kinase 1 (sFLT1), and a lack of upregulation of l-kynurenine during pregnancy compared with controls. Replacement of l-kynurenine was sufficient to rescue the abnormal placentation, reduce sFLT1 levels, and reverse the preeclampsia-like state in UNx mice.

## Results

### Blood pressure and renal phenotypes in UNx mice.

**Figure 1:**
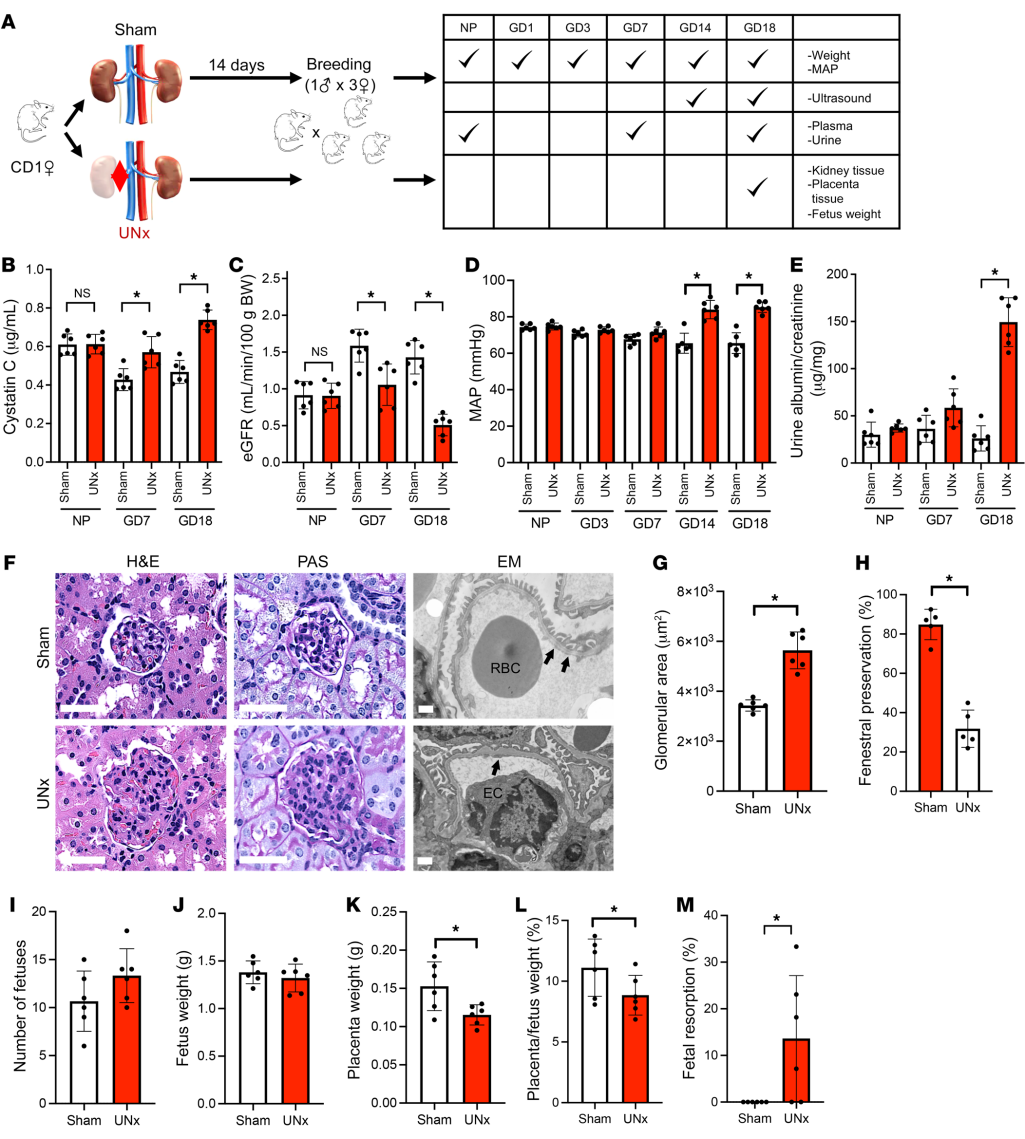
UNx mice develop a preeclampsia-like state. (**A**) Schematic representation of the experimental design. NP measurements were performed 14 days after control (Sham) or uninephrectomy (UNx) surgery and before breeding. (**B**) Plasma cystatin C measurements in sham and UNx mice. (**C**) The eGFR was calculated in sham and UNx mice as described in Methods. (**D**) MAP measurements in sham and UNx mice. (**E**) Urine albumin/creatinine ratio for sham and UNx mice. (**F**) Histopathological analysis of renal tissue from 1 representative sham mouse and 1 UNx mouse at GD18. The H&E-stained image shows large glomeruli with PAS^–^ swollen endocapillary cells in the glomeruli from UNx versus sham mice (scale bars: 50 μm). EM images of glomeruli from a sham mouse show open capillary loops containing RBCs with well-preserved endothelial fenestration (arrows), whereas glomeruli from a UNx mouse show a capillary loop containing mildly enlarged ECs and segmental loss of fenestration (arrows) (scale bars: 1 μm). (**G**) The glomerular capillary area at GD18, quantified using ImageJ as described in Methods, was increased in UNx mice compared with sham mice. (**H**) Summary data (quantified using ImageJ) for the preservation of endothelial fenestrae in glomerular capillaries from sham and UNx mice at GD18. *n* = 5 per group. Obstetrical outcomes for sham and UNx mice were assessed according to the number of fetuses (**I**), fetus (**J**) and placenta (**K**) weights, placenta/fetus weight ratio (**L**), and fetal resorption rate (**M**). Data are presented as the mean ± SD. *n* = 6 per group unless otherwise indicated. *P < 0.05 for UNx versus sham mice, by unpaired, 2-tailed Student’s *t* test.

To mimic kidney donation, we created a surgical model of UNx mice and studied their pregnancy phenotypes. Littermates that underwent sham surgery as described in Methods were used as controls (Figure 1A). Renal function was evaluated by measuring plasma cystatin C concentrations and estimating the GFR using a cystatin C–based equation, which we validated in comparison with the GFR directly measured by fluorescein plasma clearance (Supplemental Figure 1A; supplemental material available online with this article; https://doi.org/10.1172/JCI158346DS1). Plasma creatinine concentrations were found to be a less sensitive indicator of decreased GFR in mice, so only the cystatin C–based eGFR was used for the remainder of these studies (Supplemental Figure 1B). The changes in plasma cystatin C concentrations and the estimated GFR (eGFR) following uninephrectomy and prior to breeding are shown in Supplemental Figure 1, C and D. By gestational day 7 (GD7) of pregnancy, the control animals showed a 30% decrease in plasma cystatin C concentrations and a 77% increase in eGFR compared with prepregnant values, confirming the expected physiologic changes in kidney function during pregnancy in mice with both kidneys (for cystatin C: 0.43 ± 0.06 vs. 0.61 ± 0.06 μg/mL, *P* < 0.01; for eGFR: 1.59 ± 0.22 vs. 0.91 ± 0.19 mL/min/100 g body weight, *P* < 0.01) (Figure 1, B and C). Although there were no significant differences in eGFR between the UNx and control mice 2 weeks after surgery, the UNx mice showed no eGFR upregulation during pregnancy (eGFR in UNx mice increased 16% ± 18% by GD7 compared with the prepregnant state vs. a 77% ± 30% increase in control mice during the same period, *P* < 0.01) (Figure 1, B and C). Body and kidney weights in the pregnant UNx and control mice are shown in Supplemental Figure 1, E and F. These decreases in renal function in the UNx mice during pregnancy were associated with new-onset hypertension (Figure 1D) and moderate albuminuria (Figure 1E). Furthermore, histology of the kidneys from UNx mice confirmed glomerular endothelial damage and loss of endothelial fenestrae that were reminiscent of what is seen in women with preeclampsia (11) (Figure 1, F–H). In addition, staining for glomerular CD34, a marker of glomerular capillary endothelial injury (12–14), was significantly attenuated in the UNx mice when compared with controls (Supplemental Figure 2A). Other features sometimes seen in severe preeclampsia such as elevated liver enzymes were not altered (Supplemental Figure 2, B and C). We also measured whole-body plasma volumes using the Evans blue dilution method and confirmed that UNx mice had no increase in plasma volume at GD14 and GD18 compared with controls (Supplemental Figure 2, D and E). With regard to pregnancy phenotypes, we noted no significant changes in fetal weights or litter size in UNx pregnant animals, however, we observed a significantly increased rate of fetal resorption and decreased placental/fetal weight ratio in the UNx mice compared with controls (Figure 1, I–M), consistent with placental insufficiency. In sum, these data suggest that during pregnancy, UNx mice failed to show GFR upregulation and developed preeclampsia-like phenotypes in late gestation that resembled the nonsevere preeclampsia phenotypes observed during pregnancy in human kidney donors (6).

### Placental and uterine blood flow changes in UNx mice.

**Figure 2:**
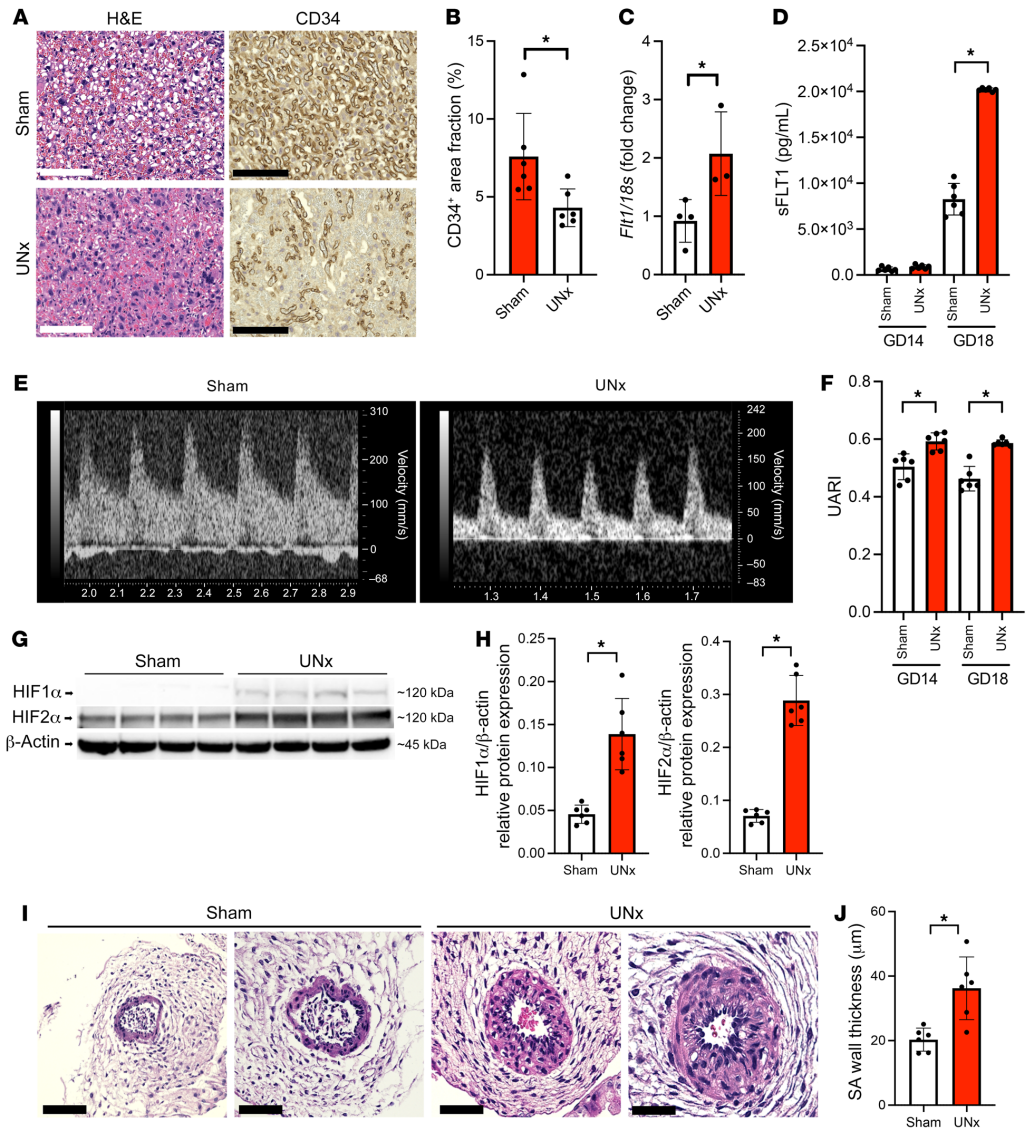
Placental and uterine blood flow changes in UNx mice. (**A**) Histopathological analysis of placental tissue from 1 representative control (sham) mouse and 1 UNx mouse at GD18 (scale bars: 125 μm). H&E-stained images show a collapsed labyrinthine vasculature in the UNx mouse compared with the sham-treated mouse. CD34 IHC confirmed the loss of labyrinthine vasculature in the UNx group. (**B**) Summary data for the CD34^+^ labyrinthine area quantified using ImageJ in sham and UNx mice at GD18. (**C**) Quantification of placental *sFlt1* mRNA expression in sham versus UNx mice at GD18. *n* = 4 per group. All data were normalized to *18s* (ΔCt). (**D**) ELISA was performed to measure sFLT1 protein expression in plasma samples from sham and UNx mice at GD14 and GD18. (**E**) Representative Doppler waveforms of uterine artery flow velocity at GD18 in 1 sham mouse and 1 UNx mouse. (**F**) The UARI calculated at GD14 and GD18 for sham and UNx mice as described in Methods. (**G**) Western blot analysis of HIF1α and HIF2α levels in placental tissue from sham and UNx mice at GD18. (**H**) Summary quantitative data for HIF1α and HIF2α protein levels normalized to β-actin expression. (**I**) Two representative photomicrographs of transversal sections of decidual SAs from sham and UNx mice at GD14 (scale bars: 125 μm). H&E-stained images show impaired SA remodeling in UNx mice compared with sham mice. (**J**) Summary data for SA wall thickness, measured as described in Methods for the sham and UNx groups. Data are presented as the mean ± SD. *n* = 6 per group unless otherwise indicated. *P < 0.05 for UNx versus sham-operated mice, by unpaired 2-tailed Student’s *t* test.

To seek evidence of abnormal placentation, we studied placentas from UNx mice and compared them with those from control mice. On H&E stains, the labyrinthine vasculature appeared collapsed (Figure 2A), and CD34 immunostaining of endothelial cells (ECs) confirmed reduced vasculature in placentas from UNx mice (Figure 2, A and B). In UNx mice, these placental changes were accompanied by a 2-fold upregulation of placental *sFlt1* mRNA expression (Figure 2C) and a 2.5-fold increase in circulating sFLT1 protein levels at GD18 (Figure 2D) that were similar to what is observed in women with preeclampsia (15). Since placental ischemia is thought to be a major driver of sFLT1 upregulation (16), we measured uterine artery blood flow using live ultrasound and Doppler imaging (Figure 2E). We found that uterine artery blood flow patterns in UNx mice were impaired compared with patterns in control animals, as evidenced by increases in the uterine artery resistive index (UARI) (Figure 2F). Interestingly, uterine artery flow abnormalities were noted in early gestation (GD14), prior to significant changes in sFLT1 concentrations. Since sFLT1 levels were not changed at GD14 (Figure 2D), these data suggest that the changes in placental perfusion were primary and that the upregulation of sFLT1 was secondary. We also measured the levels of HIF1α and HIF2α proteins, which are upregulated during ischemia and have previously been reported to be an important trigger for the upregulation of sFLT1 during preeclampsia (17). We noted that in the placentas of UNx mice, the expression of both HIF1α and HIF2α proteins was upregulated, consistent with evidence of placental ischemia (Figure 2, G and H). In addition to functional changes in uterine blood flow, we also noted structural changes in the uterine SAs, as evidenced by impaired SA remodeling that was prominent in the UNx mice, but not in the controls (Figure 2, I and J).

### Placental growth factor rescues the preeclampsia-like phenotype in UNx mice.

**Figure 3:**
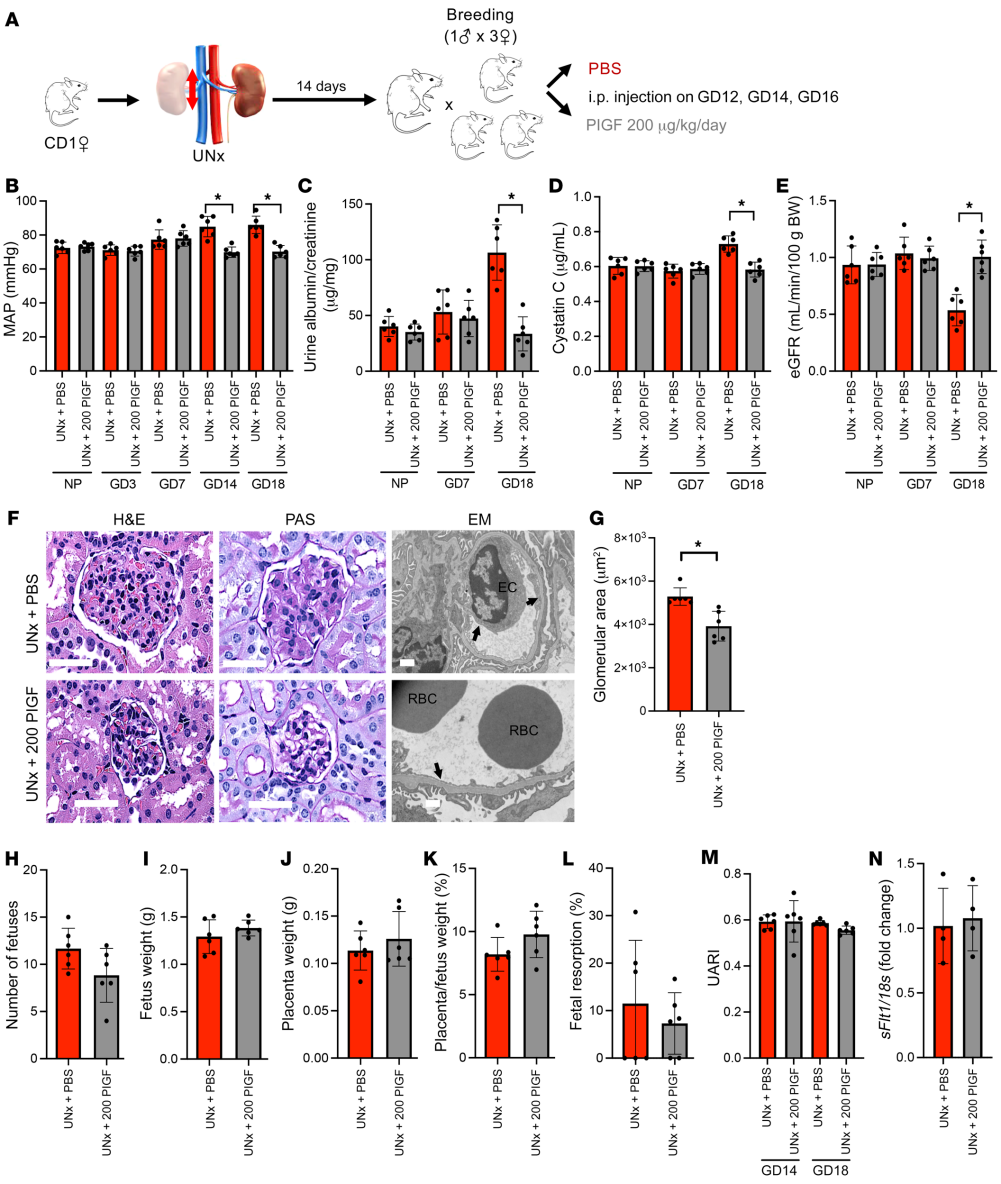
PlGF rescues the preeclampsia-like phenotype in UNx mice. (**A**) Schematic representation of the experimental design. (**B**) MAP measurements in UNx mice treated with either PBS or recombinant PlGF in the NP state and during pregnancy. (**C**) Urine albumin/creatinine ratio in UNx mice treated with PBS or PlGF. (**D**) Plasma cystatin C measurements in UNx mice treated with PBS or PlGF. (**E**) The eGFR was calculated in UNx mice treated with PBS or PlGF as described in Methods. (**F**) Histopathological analysis of renal tissue at GD18 from 1 representative UNx mouse treated with PBS and 1 UNx mouse treated with PlGF. H&E-stained images (scale bars: 50 μm) and EM analysis (scale bars: 1 μm) show significant improvement in endothelial lesions in the PlGF-treated mouse (arrow shows preserved endothelial fenestration) compared with the PBS-treated mouse (arrows show enlarged ECs with segmental loss of fenestration). (**G**) The glomerular capillary area at GD18, quantified using ImageJ as described in Methods, was decreased in UNx mice treated with PlGF compared with those treated with PBS. Obstetrical outcomes for UNx mice treated with PBS or PlGF were assessed according to the number of fetuses (**H**), fetus (**I**) and placenta (**J**) weights, placenta/fetus weight ratio (**K**), and fetal resorption rate (**L**). (**M**) UARIs were calculated at GD14 and GD18 for UNx mice treated with PBS or PlGF as described in Methods. (**N**) Quantification of placental *sFlt1* mRNA expression in UNx mice treated with PBS or PlGF at GD18. *n* = 4 per group. All data were normalized to *18s* (ΔCt). Data are presented as the mean ± SD. *n* = 6 per group unless otherwise indicated. *P < 0.05 for UNx mice treated with PlGF versus those treated with PBS, by unpaired, 2-tailed Student’s *t* test.

Since sFLT1 has been reported to be pathogenically implicated in other models of preeclampsia (18–20), we hypothesized that sFLT1 upregulation in the UNx mice was contributing to the maternal syndrome. To test this hypothesis, we treated UNx mice with recombinant placental growth factor (PlGF), which is a natural antagonistic ligand for sFLT1 (Figure 3A and Supplemental Figure 3, A and B). As predicted, PlGF therapy rescued the maternal preeclampsia phenotype, thus decreasing blood pressure, albuminuria, and glomerular endothelial damage (Figure 3, B–G, and Supplemental Figure 3, C and D). PlGF therapy was also associated with a trend toward increased fetal and placental weights, however, these findings were not significant (Figure 3, H–L). Furthermore, recombinant PlGF did not significantly affect placental perfusion (Figure 3M), placental histology (Supplemental Figure 3, E and F), or placental *sFlt1* expression (Figure 3N), suggesting that PlGF works by neutralizing late pregnancy rises in sFLT1 and improving maternal vascular health, but does not influence the uninephrectomy-associated placental abnormalities that likely initiate earlier in pregnancy. Taken together with prior data showing that sFLT1 overexpression was sufficient to induce maternal signs and symptoms of preeclampsia (21), these data suggest that sFLT1 upregulation occurred as a consequence of impaired placental perfusion and was subsequently responsible for inducing the maternal hypertension and preeclampsia-like phenotype noted in UNx mice.

### Plasma metabolite signatures during pregnancy in UNx mice.

**Figure 4:**
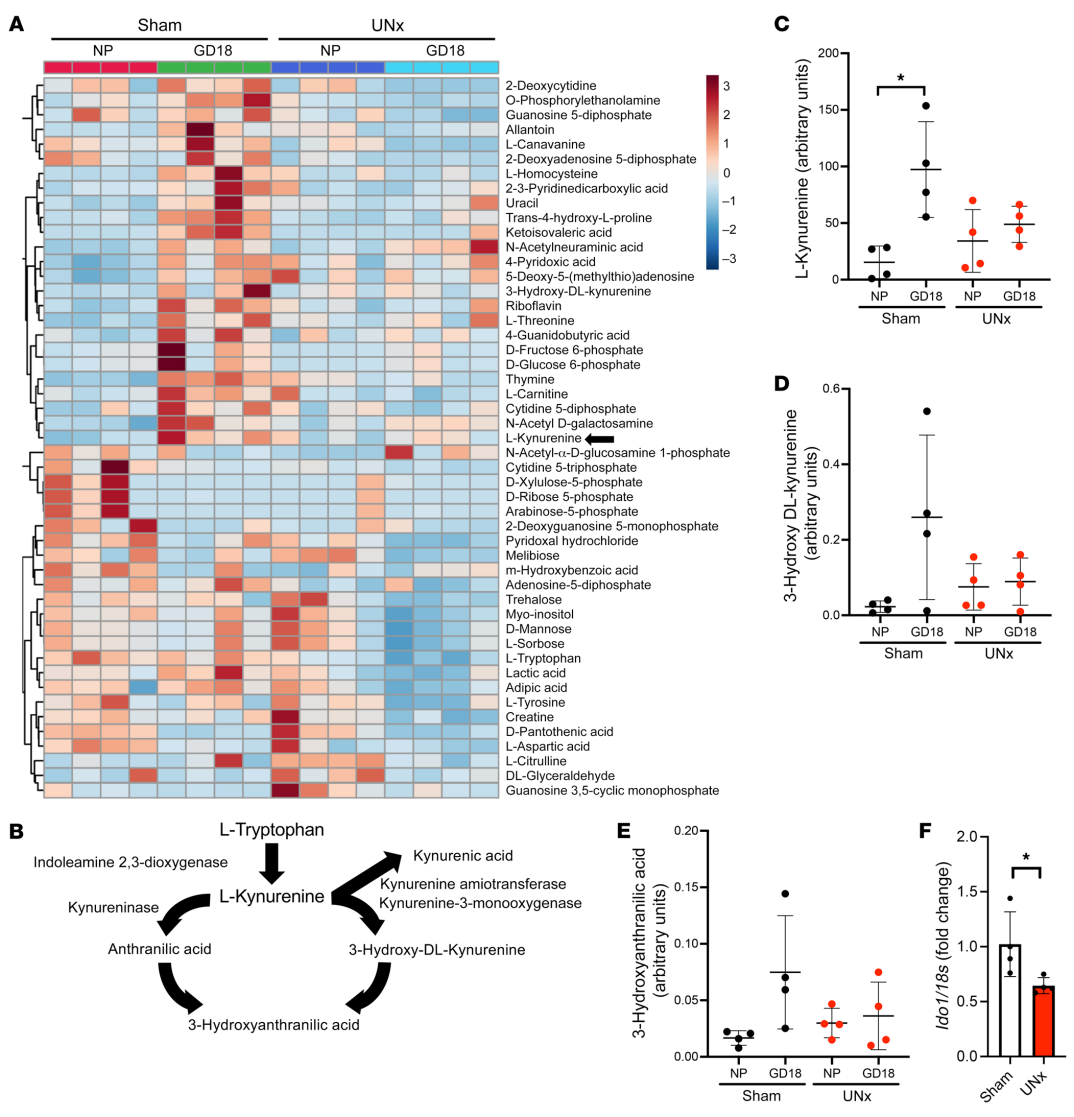
l-kynurenine and downstream metabolites are upregulated during pregnancy in sham-treated but not UNx mice. (**A**) Heatmap of metabolite changes in control and UNx mice in the NP state and at GD18, including changes in l-kynurenine (arrow). Plasma samples corresponding to the NP state were drawn 14 days after sham or uninephrectomy surgery. (**B**) Schematic of the l-tryptophan/l-kynurenine pathway. Levels of l-kynurenine (**C**), 3-hydroxy-DL-kynurenine (**D**), and 3-hydroxyanthranilic acid (**E**) in the NP state and at GD18 in sham-operated and UNx mice. Results are shown as the normalized peak intensities (arbitrary units) from the metabolite profiling studies. For each of the metabolites, the individual values, mean, and SD are shown. *n* = 4 per group. *P < 0.05, by 1-way ANOVA with Tukey’s test for multiple comparisons. (**F**) Quantification of placental *Ido1* mRNA expression in sham and UNx mice at GD18. Data are presented as the mean ± SD. *n* = 4 per group. *P < 0.05 for UNx versus sham mice, by unpaired, 2-tailed Student’s *t* test.

To evaluate potential mechanisms of how impaired renal reserve in UNx mice leads to abnormal placentation, we studied plasma metabolite profiles during pregnancy using the mass spectrometry–based dynamic multiple reaction monitoring (dMRM) assay (Agilent Technologies) described in Methods. We observed several interesting metabolite changes associated with normal pregnancy and pregnancy in UNx animals (Figure 4A and Supplemental Table 1). In order to discover potential contributors to abnormal placentation, we focused on metabolite changes associated with pregnancy that were altered in UNx animals. Several metabolites such as l-kynurenine increased during pregnancy in non-nephrectomized animals but were upregulated to a lesser degree in pregnant UNx mice (Figure 4A and Supplemental Figure 4). We focused our analyses on metabolites that are part of the l-tryptophan/l-kynurenine pathway (Figure 4B), as l-tryptophan is metabolized into l-kynurenine in the placenta by indolamine 2,3-dioxygenase (IDO), and this pathway has previously been reported to play a central role in promoting immune tolerance and vascular remodeling at the placental maternal-fetal interface (22, 23). Interestingly, several metabolites of l-tryptophan downstream of IDO, including l-kynurenine, 3-hydroxy-DL-kynurenine, and 3-hydroxyanthranilic acid, were upregulated during pregnancy in the control animals but were upregulated to a lesser extent in the pregnant UNx mice (Figure 4, C–E). Interestingly, 5 hydroxy indole acetic acid, another metabolite of l-tryptophan, was not altered (Supplemental Figure 4F), suggesting that the major impairment in UNx mice was a relative depletion of l-kynurenine and its downstream metabolites such as 3-hydroxyanthranilic acid. To explain why l-kynurenine levels were relatively decreased in the UNx mice, we measured placental *Ido1* mRNA expression and noted that UNx mice had suppressed *Ido1* mRNA levels when compared with expression levels in controls (Figure 4F).

### l-kynurenine replacement rescues the impaired placentation and preeclampsia-like phenotype in UNx mice.

**Figure 5:**
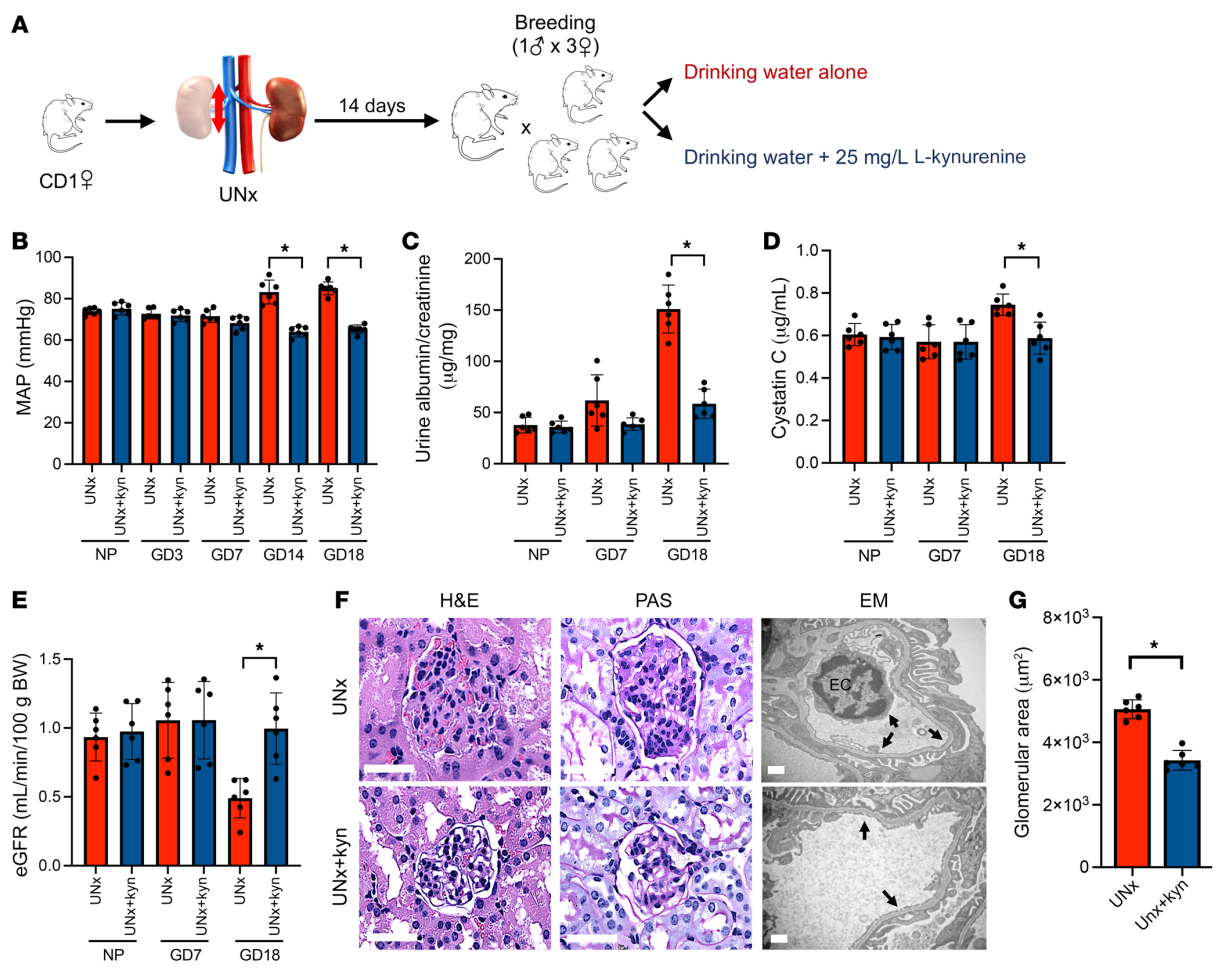
l-kynurenine supplementation rescues the preeclampsia-like phenotype in UNx mice. (**A**) Schematic representation of the experimental design. (**B**) MAP measurements in UNx mice and UNx mice treated with l-kynurenine (25 mg/L) added to drinking water (UNx+kyn) in the NP state and during pregnancy. (**C**) Urine albumin/creatinine ratio for UNx and UNx+kyn mice. (**D**) Plasma cystatin C measurements in UNx and UNx+kyn mice. (**E**) The eGFR was calculated for UNx and UNx+kyn mice as described in Methods. (**F**) Histopathological analysis of renal tissue at GD18 from 1 representative UNx mouse and 1 UNx+kyn mouse. H&E-stained images (scale bars: 50 μm) and EM analysis (scale bars: 1 μm) show a normalized glomerulus structure in the UNx+kyn mouse (arrows show preserved endothelial fenestration) compared with the UNx mouse (arrows show enlarged ECs with segmental loss of fenestration). (**G**) The glomerular capillary area at GD18, quantified using ImageJ as described in Methods, was decreased in UNx+kyn mice compared with UNx mice. Data are presented as the mean ± SD. *n* = 6 per group for all experiments. *P < 0.05 in UNx versus UNx+kyn mice, by unpaired, 2-tailed Student’s *t* test.

**Figure 6:**
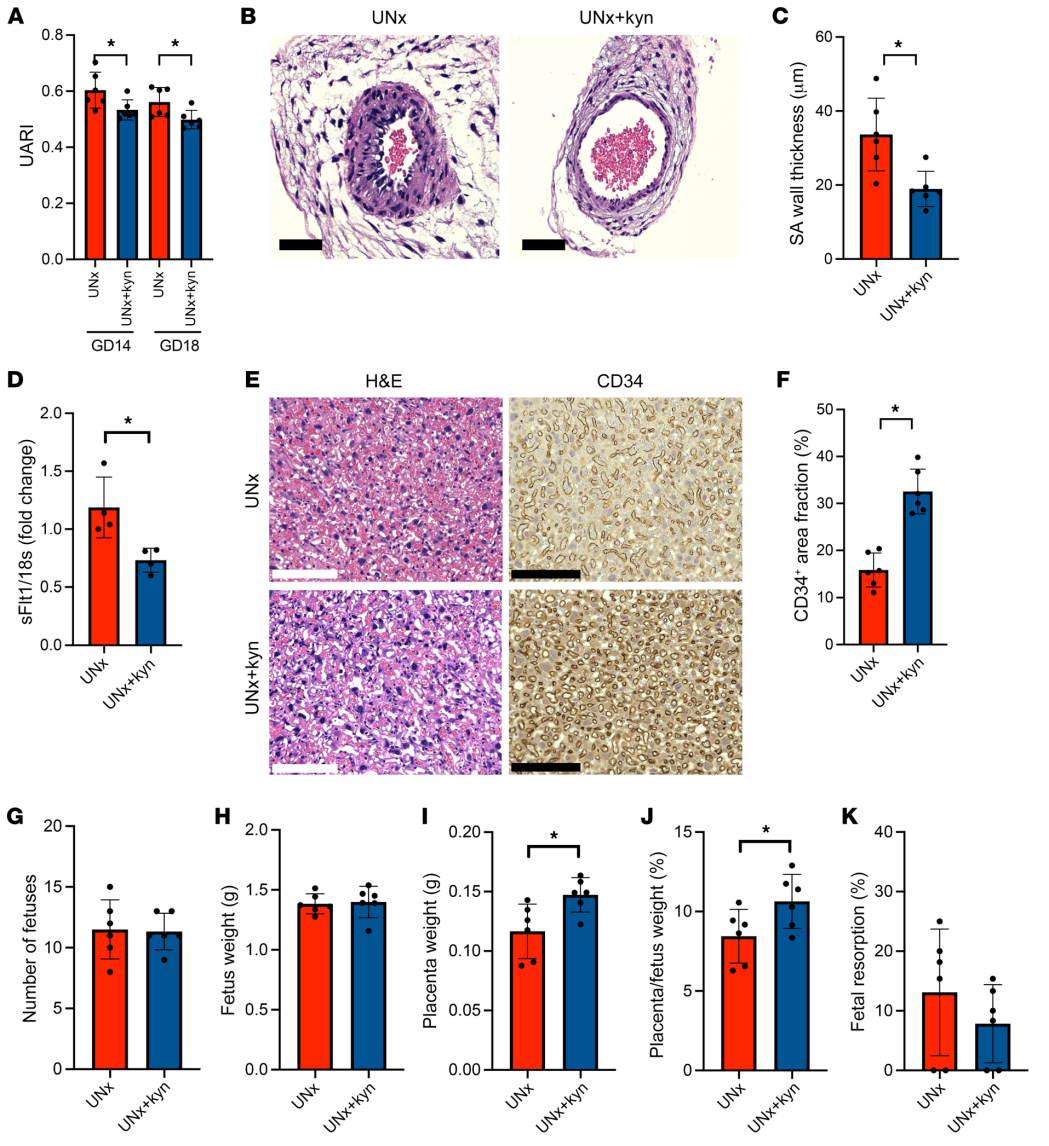
l-kynurenine supplementation rescues placental vasculature and obstetrical outcomes in UNx mice. (**A**) Summary data for UARIs at GD14 and GD18 for UNx mice and UNx+kyn mice, as described in Methods. (**B**) Representative photomicrographs of transverse sections of decidual SAs from UNx and UNx+kyn mice at GD14 (scale bars: 125 μm). SA remodeling was rescued in UNx+kyn mice as shown by H&E staining. (**C**) Summary data for SA wall thickness, measured as described in Methods, in UNx mice and UNx+kyn mice. (**D**) Quantification of placental *sFlt1* mRNA expression in UNx and UNx+kyn mice at GD18. *n* = 4 per group. All data were normalized to *18s* (ΔCt). (**E**) Histopathological analysis of placental tissue from 1 representative UNx mouse and 1 UNx+kyn mouse at GD18. H&E-stained images show normalized labyrinthine vasculature in placental tissue from the UNx+kyn mouse compared with that from the UNx mouse (scale bars: 125 μm). CD34 IHC confirmed the rescue of labyrinthine vasculature in the UNx+kyn mice. (**F**) Summary data for the CD34^+^ labyrinthine area quantified using ImageJ for UNx and UNx+kyn mice at GD18. Obstetrical outcomes for UNx and UNx+kyn mice were assessed by the number of fetuses (**G**), fetus (**H**) and placenta (**I**) weights, placenta/fetus weight ratio (**J**), and fetal resorption rate (**K**). Data are presented as the mean ± SD. *n* = 6 per group unless otherwise indicated. *P < 0.05 in UNx+kyn versus UNx mice, by unpaired, 2-tailed Student’s *t* test.

Since l-tryptophan metabolites have been previously shown to be important for early placentation and vascularization (22, 23), and because l-kynurenine has been shown to vasodilate myometrial resistance arteries (24) and improve renal function (25), we evaluated whether the replacement of l-kynurenine during pregnancy may reverse the placental flow abnormalities noted in UNx mice (Figure 5A and Supplemental Figure 5, A and B). Interestingly, l-kynurenine treatment rescued the late pregnancy preeclampsia-like phenotype in UNx mice (Figure 5, B–G, and Supplemental Figure 5, C and D). Furthermore, UNx mice fed l-kynurenine demonstrated improved placental perfusion (Figure 6A) and had normalized SA development compared with UNx mice fed drinking water alone (Figure 6, B and C). Importantly, l-kynurenine treatment also decreased placental *sFlt1* mRNA levels (Figure 6D) and was associated with improved labyrinthine vasculature (Figure 6, E and F). Last, improvements in placental perfusion in l-kynurenine–treated animals were associated with improved placental weights and reversal of placental insufficiency as shown by an increase in the placental/fetal weight ratio (Figure 6, G–K).

**Figure 7:**
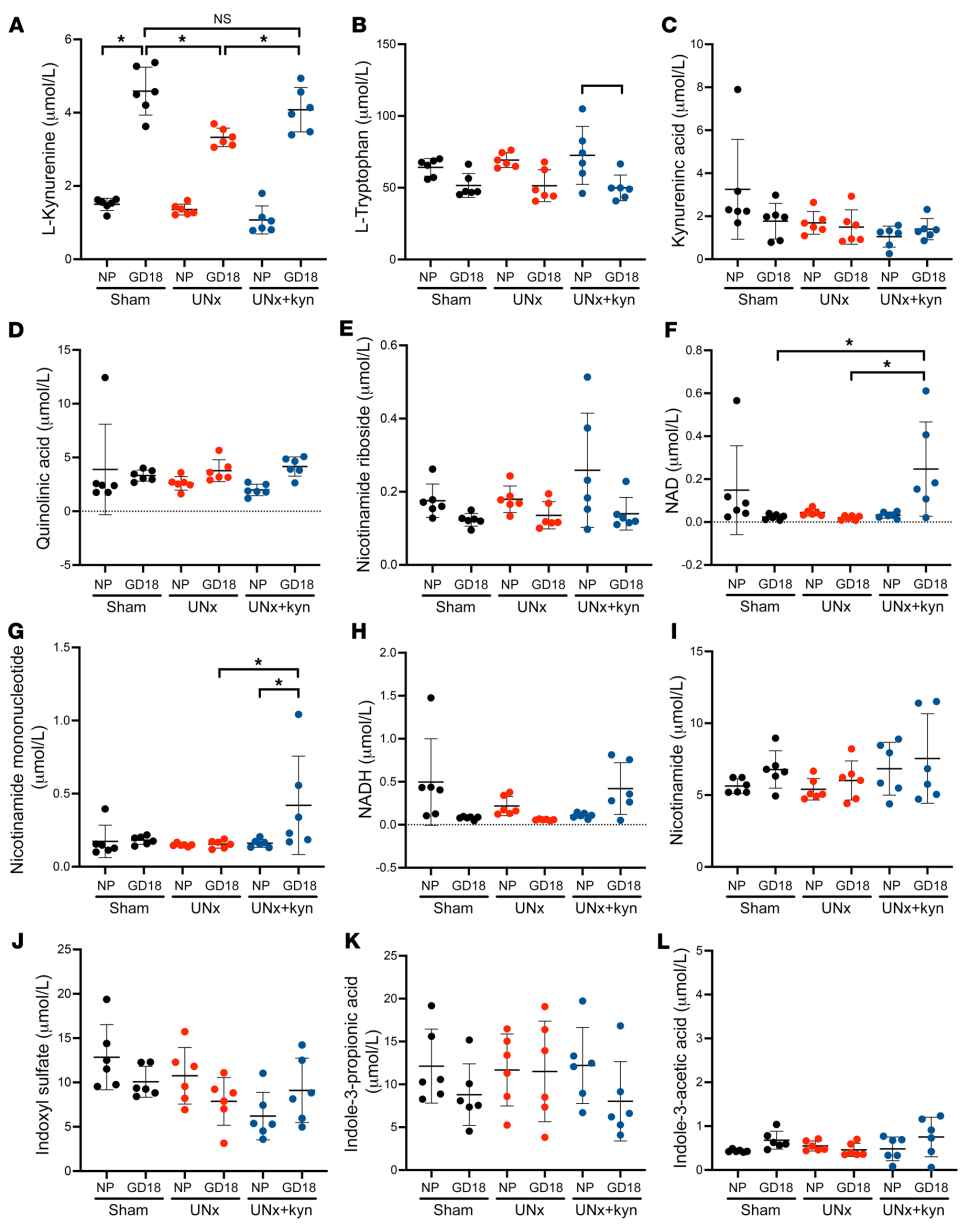
Quantitative levels of l-kynurenine and related metabolites by ID-MS/MS in sham-treated mice, UNx mice, and UNx+kyn mice. Levels of l-kynurenine (**A**), l-tryptophan (**B**), kynurenic acid (**C**), quinolinic acid (**D**), nicotinamide riboside (**E**), NAD (**F**), nicotinamide mononucleotide (**G**), NADH (**H**), nicotinamide (**I**), indoxyl sulfate (**J**), indole-3-propionic acid (**K**), and indole-3-acetic acid (**L**) in the NP state and at GD18 in control, UNx, and UNx+kyn mice, as quantified by ID-MS/MS. The mean and SD of individual values are depicted for each of the metabolites. *n* = 6 per group. *P < 0.05, by 1-way ANOVA with Tukey’s test for multiple comparisons.

To confirm relative l-kynurenine depletion in UNx mice compared with controls during pregnancy and the restoration of l-kynurenine levels in UNx mice treated with l-kynurenine, we used a second quantitative assay platform (isotope-dilutional tandem mass spectrometry [ID-MS/MS], see Supplemental Methods) to measure l-kynurenine levels and related metabolites. Consistent with the metabolite screening data (Figure 4, A and C), we noted that l-kynurenine levels increased approximately 3-fold from the NP state to late pregnancy in control animals (Figure 7A). In contrast, l-kynurenine concentrations were approximately 30% lower at GD18 in UNx mice when compared with controls, corroborating the effects of uninephrectomy on l-kynurenine production in pregnancy (Figure 7A). We also noted a pattern of decreased l-tryptophan concentrations among all 3 groups of GD18 mice compared with their NP levels, although the difference was only statistically significant among the UNx mice (Figure 7B); this suggests that l-tryptophan utilization was increased during pregnancy, perhaps partly by metabolism of l-tryptophan to l-kynurenine. As expected, l-kynurenine treatment in drinking water during pregnancy in UNx mice led to increased circulating levels of l-kynurenine that were similar to levels noted in control pregnant mice (4.1 ± 0.6 μmol/L vs. 4.6 ± 0.7 μmol/L, *P* = NS). Interestingly, kynurenic acid and quinolinic acid, both metabolites of l-kynurenine, were not altered (Figure 7, C and D). However, l-kynurenine treatment resulted in increases in multiple metabolites of the NAD^+^ salvage pathway (NAD^+^ and nicotinamide mononucleotide), whereas nicotinamide and nicotinamide riboside levels were unaltered (Figure 7, E–I). In order to test whether there were changes associated with uninephrectomy or pregnancy in l-tryptophan metabolites that are not part of the l-kynurenine/IDO pathway, we measured indoxyl sulfate, indole-3-propionic acid, and indole-3-acetic acid levels, but found that these were also not significantly altered (Figure 7, J–L). Together, these findings suggest that l-tryptophan metabolism to l-kynurenine by the IDO enzyme is normally upregulated in pregnancy but is constrained in animals after uninephrectomy. Furthermore, the selective increases in NAD^+^ and nicotinamide mononucleotide (without increases in kynurenic acid) in mice treated with l-kynurenine suggest that the increased l-kynurenine production may have been preferentially shunted toward the de novo NAD^+^ synthetic pathway.

## Discussion

In this study, we report that outbred mice with prior uninephrectomy had impaired adaptation to pregnancy that manifested as a lack of increased GFR or plasma volume during early pregnancy, followed by a subsequent nonsevere preeclampsia-like phenotype with hypertension, albuminuria, and glomerular endothelial damage. Moreover, the preeclampsia-like phenotypes during late pregnancy were accompanied by decreased placental perfusion, impaired SA remodeling, and increased placental and circulating levels of sFLT1, a protein that has been implicated as having a prominent role in the pathogenesis of preeclampsia (26). We noted an upregulation of placental HIF1α and HIF2α protein levels, which may have been a consequence of placental ischemia and potentially triggered excess sFLT1 production by the placenta in the UNx mice. The lack of SA remodeling and accompanying placental ischemia caused by uninephrectomy led to alterations in many circulating molecules; however, recombinant PlGF (18), a natural antagonist for sFLT1, was sufficient to rescue the maternal signs of preeclampsia, confirming the contribution of sFLT1 in mediating the late pregnancy maternal preeclampsia–like phenotypes in UNx animals. To evaluate upstream factors that mediate placental ischemia in UNx mice, we performed unbiased metabolite screens and noted decreases in concentrations of l-tryptophan and l-tryptophan–derived l-kynurenine. We confirmed that l-kynurenine levels rose 3-fold during pregnancy, presumably due to IDO expression in the placenta to support immune tolerance (27). Decreases in l-kynurenine concentrations in UNx mice were accompanied by a relative suppression of IDO expression in the placenta. Furthermore, replacement of l-kynurenine during pregnancy in UNx mice was sufficient to improve placental flow abnormalities and the maternal preeclampsia–like phenotype and to reverse placental insufficiency and reduce placental *sFlt1* expression. Together, these data suggest that failure to upregulate l-kynurenine during pregnancy in UNx mice due to decreased placental perfusion and suppressed IDO expression may have been the trigger for their impaired placentation, antiangiogenic state, and subsequent preeclampsia-like phenotypes.

The unfortunate association between kidney donation and an elevated risk of preeclampsia emerged from analyses of large data sets from national registries (9). In an early small case series, Davison et al. reported that although a group of 5 women carrying 1 kidney did not experience an upregulation of their GFR, there were no adverse pregnancy outcomes (28). In another study of 16 transplant recipients, the same group had relative GFR decreases in renal allograft recipients during pregnancy, and many of them developed late pregnancy gestational proteinuria, however, blood pressure data were not reported (29). In contrast to these small studies, Reisseter et al. first reported a 2-fold increase in the risk of preeclampsia (5.7% vs. 2.6%) using data from 326 donors from the Norwegian Renal Registry (8). Ibrahim et al. also confirmed a 6-fold higher risk of preeclampsia among previous living donors at a single center (*n* = 1085) when compared with nondonor pregnancy outcomes (7). Garg et al. reported a 2.4-fold greater risk of preeclampsia and a 2.2-fold higher risk of gestational hypertension in kidney donors (*n* = 85) compared with matched nondonors (*n* = 510) (6). Donor nephrectomy results in a GFR reduction of up to 30% in NP patients, although intuitively, it would be thought to reduce total renal reserve by 50%. It has been argued that impaired renal reserve leads to a lack of normal physiological adaptation and a higher risk of preeclampsia, but until now it was unknown what feto-maternal alterations downstream of renal impairment may be mediating this risk.

Why does underlying renal impairment lead to more preeclampsia? Some have hypothesized that a failure to increase the GFR and adapt to the plasma expansion of normal pregnancy may underlie preeclampsia (11), but experimental proof for this hypothesis was lacking. Kidney donors wishing to become pregnant provide a natural human experiment to determine whether decreased renal reserve and failure to upregulate GFR could contribute to the risk of preeclampsia. During pregnancy, l-tryptophan metabolism to l-kynurenine and to metabolites further downstream is upregulated due to robust placental IDO expression and may play a critical role in mediating immune tolerance and vascular remodeling at the maternal-fetal interface (22). Prior studies in mice genetically deficient in the IDO enzyme (the enzyme expressed by the placenta that is responsible for the conversion of l-tryptophan into l-kynurenine) suggest that blocking l-tryptophan conversion into l-kynurenine in the placenta could lead to impaired placentation and pregnancy complications such as preeclampsia (30). It follows that interference with the upregulation of placental l-kynurenine production during pregnancy may have significant consequences. Humans or animals with renal impairment have mild l-tryptophan deficiency, possibly due to upregulation of l-tryptophan–metabolizing TDO and IDO enzymes in the liver and other organs (31), and possibly to impaired renal l-tryptophan resorption or other mechanisms (32). During pregnancy, when l-tryptophan is used to accommodate increased gestational protein synthesis and when the placental IDO enzyme is expressed, causing placental metabolism of l-tryptophan to l-kynurenine (33), there may be further depletion of maternal circulating l-tryptophan metabolites due to an increased need to support the developing fetus. Our data suggest that preexisting renal impairment reduced the availability of l-tryptophan and thus, when combined with subsequent decreases in placental IDO enzymes (perhaps as an adaptive response to placental hypoperfusion caused by low plasma volume), resulted in a profound decrease in the production of l-kynurenine. Our data also show that replacement of l-kynurenine was sufficient to reverse the abnormal placentation and increased risk of developing preeclampsia. Taken together with epidemiological studies that have demonstrated the relationship between subnormal kidney function, abnormal placentation, and preeclampsia, our experimental findings in pregnant mice provide evidence that failure to upregulate l-kynurenine production due to a decrease in the GFR directly leads to abnormal placentation and a subsequent antiangiogenic state and preeclampsia.

Although our study provides evidence that relative l-kynurenine deficiency caused by renal impairment played a central role in mediating the risk of preeclampsia, it also raises several unanswered questions. How do renal impairment and failure to increase the GFR lead to a failure to increase plasma volume during pregnancy? What is the mechanism of continual plasma volume expansion during pregnancy despite paradoxically lower blood pressures (34)? These questions are important to understand the pathophysiology of preeclampsia, as several small studies have documented a failure of plasma volume expansion among women who subsequently develop preeclampsia (35). Some have hypothesized that nitric oxide and perhaps progesterone may be central to these seemingly paradoxical adaptations that would normally not coexist in the NP state (34). Further studies are needed to evaluate the mechanism by which l-kynurenine improves placentation. Although we did not find statistically significant changes in nicotinamide or NAD^+^ levels in UNx mice when compared with mice with 2 kidneys, l-kynurenine treatment led to upregulation of NAD^+^ and nicotinamide mononucleotide (precursor of NAD^+^), suggesting that treatment led to preferential upregulation of the de novo pathway for NAD^+^ synthesis. Prior work has suggested that nicotinamide therapy rescued 2 mouse models of preeclampsia (36). Future studies should explore the de novo NAD^+^ synthetic pathway in UNx mice in detail and evaluate whether the beneficial effects of l-kynurenine therapy are direct via its own receptor (37, 38) or indirect via the upregulation of tissue NAD^+^ levels (25, 39). The accumulation of gut microbiome–derived l-tryptophan metabolites (like indoxyl sulfate) in patients with kidney disease has been well established and reported to cause harm (40). However, we did not find any significant changes in indoxyl sulfate in our study, suggesting that, at least in these animals with mild renal insufficiency, the altered l-tryptophan–derived metabolites were largely specific to metabolites downstream of the IDO pathway. In non–l-tryptophan pathways, we also identified several metabolites, such as l-homocysteine and l-carnitine (Figure 4A, Supplemental Figure 4, and Supplemental Table 1), that failed to upregulate during pregnancy when compared with levels in control animals. It would be important to know whether any of these metabolites also play a role in the physiological volume expansion of pregnancy and placentation. Baylis and Wilson reported that in chronically UNx rats, the effects of multiple consecutive pregnancies did not lead to any deleterious long-term effects on the glomeruli, however, the phenotypes during pregnancy were not assessed (41). We noted that in our outbred CD1 strain of mice, the preeclampsia phenotypes observed in UNx mice were not severe, but consistent and uniform. Whether this was related to impaired renal vasodilatory capacity, as previously described in aged CD1 mice (42), or whether the background genetic variability had a stabilizing effect on the phenotypic endpoints (43) remains unknown.

It is important to note that, in our study, we found evidence supporting a role for the antiangiogenic factor sFLT1 in the development of the preeclampsia-like state in our nephrectomized animals, but our data suggest that this effect happened late in pregnancy, after failure to increase the GFR and poor placentation. The antiangiogenic factor sFLT1, which antagonizes circulating bioavailable VEGF and PlGF, is significantly elevated in women with preeclampsia (44) and is thought to be specifically upregulated as a result of placental ischemia (16). Upregulated sFLT1 during preeclampsia is not an epiphenomenon; in our current animal model of UNx mice, which developed a preeclampsia-like state during late-term pregnancy, and in prior experimental models of preeclampsia in rats and baboons, neutralization of circulating sFLT1 with its natural antagonistic ligand PlGF or with RNA interference was effective in ameliorating the signs and symptoms of preeclampsia (18, 19). The findings reported here may thus be added to the list of studies that confirm a role for sFLT1 in the pathogenesis of the maternal syndrome of preeclampsia.

We believe the implications of our study are profound for young women willing to donate a kidney and who also plan to have a child or children in the future, as they need to be counseled regarding the possible risk of adverse pregnancy outcomes. Furthermore, women with subclinical or undiagnosed renal impairment may also be at risk (2). Our experimental findings suggest that this risk is mediated by impaired placentation that leads to sFLT1 upregulation. If l-kynurenine deficiency is a central mediator of this risk, it would be important to study women who become pregnant to confirm that they are not l-tryptophan deficient and have appropriate upregulation of l-kynurenine levels, and to determine whether kidney donors or women with renal impairment of other causes could benefit from supplementation with l-kynurenine, l-tryptophan, or related amino acids to enhance placentation and improve pregnancy outcomes. Since sFLT1 levels rise approximately 5 weeks before the onset of clinical disease (44), kidney donors who choose to become pregnant could also undergo serial plasma sFLT1 monitoring throughout pregnancy to determine the risk and timing of the onset of preeclampsia. In sum, using mouse models, we provide mechanistic evidence for how impaired renal reserve may contribute to the risk of preeclampsia. Humans and mice both have hemochorial placentation and share many structural similarities. Comparative systems biology studies suggest that more than 80% of the proteins and pathways known to cause placental phenotypes in mice are conserved in humans (45). Nevertheless, follow-up studies will be needed to evaluate whether changes in l-kynurenine and related metabolites in women with impaired renal reserve will correlate with adverse pregnancy outcomes. 

## Methods

### Animals.

Since outbred mice tend to be better breeders than inbred mice and our aim was to assess pregnancy physiology in diverse group of animals, we chose outbred mice for our studies. Eight-week-old female and male CD-1 outbred mice were purchased from Charles River Laboratory. Animals were housed in a barrier- and pathogen-free animal facility with a 12-hour light/12-hour dark cycle and were fed a regular chow diet.

### Uninephrectomy model.

Surgery was performed in 8-week-old female mice. All mice were randomly assigned to undergo either sham surgery or uninephrectomy. Mice were anesthetized with isoflurane and then placed on the left lateral side on a warming plate to maintain body temperature at 37°C. After anesthesia induction and before the start of the surgical procedures, carprofen (5 mg/kg) and buprenorphine (0.1 mg/kg) were injected subcutaneously. The surgical site was aseptically prepped by removing the hair and then disinfected with chlorhexidine followed by alcohol. A right lateral incision was made to expose and then free the animal’s right kidney from the surrounding tissue. The kidney was gently pulled out from the incision, and the adrenal gland was freed and replaced into the abdominal cavity. In UNx animals, the renal blood vessels and the ureter were ligated with a 7-0 suture; the kidney was then removed by transecting the vessels and ureter just distal to the ligature. In sham animals, the kidney was simply left in the abdominal cavity. The muscle incision was closed with a 6-0 suture, and then the skin incision was closed with a 5-0 nonresorbable nylon suture, and Vetbond tissue adhesive (3M Health Care) was added. Triple antibiotic ointment was applied to the wound site. At 10 weeks of age (2 weeks after uninephrectomy or sham surgery), the female mice were housed with the male mice to generate pregnant mice. The presence of plug was considered as GD1. Pregnant mice were then weighed on GD1, GD3, GD7, GD14, and GD18 to confirm progression of the pregnancy and monitored for maternal and fetal phenotypes of preeclampsia as described below.

In a separate study, mice with a prior uninephrectomy were assigned to receive either recombinant PlGF (200 μg/kg body weight) that was synthesized as described elsewhere (18) or sterile PBS via intraperitoneal injection on GD12, GD14, and GD16. To assess a protective role for l-kynurenine, we also studied UNx mice receiving l-kynurenine in their drinking water at 25 mg/L throughout pregnancy (GD1–GD18) and compared their pregnancy phenotypes with UNx mice receiving drinking water during pregnancy.

### Maternal preeclampsia phenotype characterization.

Systolic blood pressure (SBP) and diastolic blood pressure (DBP) were measured from the mouse tail with a CODA noninvasive plethysmography blood pressure transducer (Kent Scientific) in the NP state (2 weeks after sham or uninephrectomy surgery and prior to breeding) and at GD1, GD3, GD7, GD14, and GD18. Mean arterial pressure (MAP) was calculated as DBP + 1/3 pulse pressure and reported as the mean ± SD as described elsewhere (46).

To measure renal function and other biomarkers, blood samples were collected using EDTA tubes via submandibular puncture in the NP state (2 weeks after sham or uninephrectomy and prior to breeding) and then during pregnancy at GD7, GD14, and GD18. Plasma was separated by centrifugation and then stored at –20°C for subsequent analysis. Plasma creatinine and cystatin C concentrations were determined using a creatininase assay (Diazyme) and a Quantikine ELISA Mouse/Rat Cystatin C Immunoassay (R&D Systems), respectively. We calculated the eGFR using the cystatin C–based equation [GFR (mL/min/100 g body weight) = –3.2886 × plasma cystatin C (μg/mL) + 2.9409], which was first validated by directly measuring the GFR and cystatin C and creatinine levels in a group of mice with varying degrees of renal insufficiency (Supplemental Figure 1, C and D). Plasma sFLT1 levels were measured using the Mouse VEGFR1/Flt-1 Quantikine ELISA Kit (R&D Systems) as described previously (21). To measure proteinuria, urine samples were collected 2 weeks after nephrectomy and during pregnancy at GD7, GD14, and GD18 and then stored at –20°C for subsequent analysis. Urine albumin excretion was measured using the Albuwell M ELISA kit (Ethos Biosciences) and normalized to urine creatinine excretion, measured using the Creatinine Companion assay (Ethos Biosciences) according to the manufacturer’s instructions. Results are expressed as micrograms of albumin per milligram creatinine.

### Uterine artery Doppler and fetal studies.

Uterine artery Doppler studies were performed at GD14 and GD18. Mice were anesthetized with isoflurane and placed in the supine position on a heated stage to maintain body temperature at 37°C. Fur was removed from the abdominal area using depilatory cream. A Vevo 3100 FujiFilm Ultrasonography Apparatus (Visual Sonics) was used to identify the left uterine artery and measure flow velocity (V) in the “pulse wave Doppler” mode. The UARI was then calculated as (systolic velocity – diastolic velocity)/systolic velocity.

Mice were euthanized at GD18, and pups were harvested from the uterus along with the placenta. Each pup and corresponding placenta were weighed. Resorption rates were calculated as the number of dead embryos/the number of healthy embryos + the number of dead embryos.

### Kidney and placenta histology.

Left kidneys were removed immediately after the mouse was sacrificed, and the kidney was weighed and gently cut into separate tissue fragments. One fragment was placed into a 4% paraformaldehyde solution for subsequent optical microscopy analysis. Staining was performed with H&E and periodic acid–Schiff (PAS) colorations. For glomerular area quantification, 20 glomeruli per mice were located from paraformaldehyde-fixed kidney sections stained with H&E (*n* = 6 animals per group). The contours of the glomerular capillary tuft were delineated, and the corresponding area was measured using ImageJ software (NIH) as previously described (47, 48). To quantify CD34^+^ ECs among the glomerular capillaries, IHC was performed to stain for CD34 (rabbit monoclonal anti-CD34, 1:500, Abcam, Ab81289) with a hematoxylin counterstain. Heat-mediated antigen retrieval (95°C for 30 minutes with citrate buffer, pH 6) was performed after the deparaffinization protocol with xylene and before commencing staining for CD34. ImageJ was used to calculate the fraction of CD34^+^ areas in the mouse glomeruli (*n* = 20 glomeruli per mouse). Each plot represents the average of all images from 1 mouse (*n* = 6 mice per group). For electron microscopy (EM), a few tiny fragments of kidney tissues fixed in 3% glutaraldehyde were post-fixed in osmium tetraoxide and embedded in epoxy resin. Ultrathin sections were collected on carbon-coated formvar grids and stained with uranyl acetate and lead citrate as previously described (49). The images were obtained with a Hitachi 7700 transmission electron microscope (Hitachi). For ultrastructural evaluation of glomerular endothelial fenestrae, the images of glomerular capillary loops (at least 13 loops per mouse) were obtained from 5 sham-treated mice and 5 UNx mice. Preservation of the endothelial fenestrae in the glomerular capillary bed in each image was evaluated by a renal pathologist in a blinded manner and expressed from 0% (complete loss of fenestrae) to 100% (intact fenestrae). Each plot represents the average of all images from 1 mouse.

For placental histology, 3 placentas per mouse were isolated immediately after sacrifice and then gently cut into separate tissue fragments. One fragment was placed in a 4% paraformaldehyde solution for subsequent optical microscopy analysis. Staining was first performed with H&E for morphology assessment. To quantify the vasculature area within the labyrinth, we used IHC to stain for CD34 (rabbit monoclonal anti-CD34, 1:500, Abcam, ab81289) with hematoxylin counterstaining. Heat-mediated antigen retrieval (95°C for 30 minutes with citrate buffer, pH 6) was performed after the deparaffinization protocol with xylene and before commencing CD34 staining. ImageJ was used to calculate the positive fraction of the area under ×20 objective magnification.

In a subset of animals, SAs were located in the decidua at GD14 in paraformaldehyde-fixed implantation sites that were stained with H&E. The lumen circumference (LC) and wall circumference (WC) were delineated and measured under ×20 objective magnification using ImageJ. The lumen diameter (LD) was calculated from the LC as LD = LC/π, and the wall diameter (WD) was calculated as WD = WC/π. Wall thickness was then calculated as (WD – LD)/2, as previously described (50).

### Plasma volume measurements.

Plasma volume was measured in the NP state, at GD14, and at GD18 in separate groups of animals using the Evans blue dilution method (51). Evans blue dye (MilliporeSigma; 1 μL/g body weight of a solution of 0.5% w/v in sterile isotonic saline) was injected via the tail vein under isoflurane anesthesia with a 30 gauge needle. After 10 minutes, a blood sample was collected by submandibular puncture, and plasma was separated by centrifugation and then stored at –20°C for subsequent analysis. ODs of plasma samples and standard dilutions were measured at 620 nm. A standard curve was used to determine the plasma Evans blue concentration in each sample, and then the corresponding plasma volumes for each mouse as (concentration × volume of dye injected)/plasma Evans blue concentration was calculated.

### Protein extraction and Western blot analysis.

After euthanasia, placental fragments were immediately snap-frozen into liquid nitrogen, then stored at –70°C for subsequent analysis. Placental samples were lysed in lysis and extraction buffer (Thermo Fisher Scientific) containing Halt Protease inhibitor cocktail (Thermo Fisher Scientific) and PhosSTOP (Roche). Equal amounts of total protein lysates were run on a Bolt 4%–12%, Bis-Tris, 1.0 mm, Mini Protein Gel (Thermo Fisher Scientific) and transferred onto a nitrocellulose membrane (Thermo Fisher Scientific). The membrane was then incubated overnight at 4°C in 5% BSA and 0.1% Tween-20 in PBS with HIF1α (rabbit monoclonal anti–mouse HIF1α, 1:1,000, Cell Signaling Technology, 14179S), HIF2α (rabbit monoclonal anti–mouse HIF2α, 1:1,000, Cell Signaling Technology, 57921S), or β-actin (rabbit monoclonal anti–mouse β-actin, 1:1,000, Cell Signaling Technology, 8457S) antibodies. Membranes were subsequently probed with specific HRP-conjugated secondary antibodies (1:1,000) (Cell Signaling Technology) for 1 hour at room temperature. Bands were revealed using iBright FL1000 (Thermo Fisher Scientific) and quantified using densitometric scanning with ImageJ. For each animal, the mean relative expression of HIF1α and HIF2α was calculated for at least 3 separate placentas. HIF1α and HIF2α expression levels were normalized to β-actin expression.

### RNA extraction and real-time quantitative PCR.

After sacrifice, placental tissue was placed in 1 mL RNAlater (Invitrogen, Thermo Fisher Scientific) and then stored at –70°C for subsequent analysis. The total RNA was extracted from each sample with TRIzol Reagent (Invitrogen, Thermo Fisher Scientific), and their concentrations were determined using a NanoDrop One (Thermo Fisher Scientific) spectrophotometer. To perform reverse transcription (RT), 1 μg RNA sample was added to 2 μL 10× RT buffer, 0.8 μL 25× dNTmix, 2 μL 10× random primers, 1 μL multiscribe RT, and 1 μL RNase inhibitor to a final reaction volume of 10 μL as recommended by the manufacturer (Applied Biosystems). TaqMan quantitative real-time PCR was performed for quantification of total *sFlt1* (all isoforms of *sFlt1* and *Flt1*, Mm00438980_m1, Thermo Fisher Scientific), *Ido1* (Mm00492590_m1, Thermo Fisher Scientific), and *18S* (Hs99999901_s1, Thermo Fisher Scientific) mRNA using the QuantiStudio3 thermocycler (Applied biosystems). Relative total *sFlt1* mRNA expression was normalized to *18S* mRNA, and fold changes were calculated using the 2^–ΔΔ^Ct method.

### Metabolomics analysis.

Metabolomics studies were performed as previously described (52, 53) using a dMRM assay targeting primarily the central carbon chain metabolites. Metabolite extractions were analyzed with an Agilent 6470A triple quadrupole mass spectrometer, operating in negative ion mode, connected to an Agilent 1290 Ultra High-Performance Liquid Chromatography (UHPLC) system and utilizing the MassHunter Metabolomics dMRM Database and Method to scan for 219 polar metabolites within each sample. The method is a highly reproducible and robust ion-pair reversed-phase (IP-RP) chromatographic method developed to separate anionic and hydrophobic metabolites. Tributylamine (TBA), a volatile ternary amine amenable to electrospray ionization that functions as an ion pair reagent, was used to facilitate and improve reproducible retention of acidic metabolites. The ion-pairing liquid chromatography/mass spectrometry (LC/MS) method enables simultaneous analysis of multiple metabolite functional classes, including amino acids, citric acid cycle intermediates and other carboxylic acids, nucleobases, nucleosides, phosphosugars, and fatty acids. Mobile phases consisted of HPLC- or LC/MS-grade reagents. Buffer A was water with 3% methanol, 10 mM TBA, and 15 mM acetic acid. Buffers B and D were isopropanol and acetonitrile, respectively. Finally, buffer C was methanol with 10 mM TBA and 15 mM acetic acid. The analytical column used was an Agilent ZORBAX RRHD Extend-C18 1.8 μm 2.1 × 150 mm coupled with a ZORBAX Extend Fast Guard column for UHPLC Extend-C18, 1.8 μm, 2.1 mm × 5 mm. The MRM method takes advantage of known retention time information for each compound to create MRM transition lists that are dynamically created throughout an LC/MS run using a window around the expected retention times. Resulting chromatograms were visualized in Agilent MassHunter Quantitative Analysis for QQQ. The final peaks were manually checked for consistent and proper integration. Results were analyzed in MetaboAnalyst (http://www.metaboanalyst.ca).

Direct measurements of l-kynurenine and other metabolites of l-tryptophan were performed using HPLC and ID-MS/MS as described in Supplemental Methods and Supplemental Tables 2 and 3.

### Statistics.

Data were graphed and statistics calculated using GraphPad Prism, version 9.2 (GraphPad Software). The number of mice used per experiment is indicated in the legends to figures. All comparisons between 2 groups were assessed using an unpaired, 2-tailed Student’s *t* test with a 95% CI. Data comparing 3 or more groups were analyzed using 1-way ANOVA with Tukey’s multiple-comparison test. Statistically significant differences were defined as a *P* value of less than 0.05.

### Study approval.

All animal experiments were conducted in accordance with the NIH’s *Guide for the Care and Use of Laboratory Animals* (National Academies Press, 2011), and all protocols used in this study were approved by the IACUC of Cedars-Sinai Medical Center.

## Author contributions

VD, AHB, and SAK designed the research study. VD conducted experiments. VD, MY, CH, AEC, and SA acquired data. VD and MY performed histopathology and EM studies. AHB, AS, and JEVE performed metabolomics studies. VD, AHB, BJ, RT, and SAK analyzed data. VD, AHB, and SAK wrote the first version of the manuscript. All authors contributed to drafting of the manuscript, and all authors approved the final version of the manuscript.

## Supplementary Material

Supplemental data

## Version 1 08/09/2022

In-Press Preview

## Version 2 10/17/2022

Electronic publication
