# Supplementary material for: Impaired renal reserve contributes to preeclampsia via the kynurenine and soluble fms–like tyrosine kinase 1 pathway
Source: J Clin Invest. 2022 Oct 17;132(20):e158346. doi: 10.1172/JCI158346 (PMC9566901; doi:10.1172/JCI158346)
Supplement: Supplemental data [file jci-132-158346-s085.pdf]

Supplemental Material

Impaired renal reserve contributes to preeclampsia via the kynurenine and soluble fms-like tyrosine kinase 1 pathway

Vincent Dupont[1,2], Anders H. Berg[3], Michifumi Yamashita[3], Chengqun Huang[3], Ambart E. Covarrubias[1], Shafat Ali[1], Aleksandr Stotland[4], Jennifer E. Van Eyk[4], Belinda Jim[5], Ravi Thadhani[6], and S. Ananth Karumanchi[1]

[1]Department of Medicine, Cedars-Sinai Medical Center, Los Angeles, CA

[2]EA-3801, Université de Reims Champagne-Ardenne, Reims, France

[3]Department of Pathology and Laboratory Medicine, Cedars-Sinai Medical Center, Los Angeles, CA

[4]Department of Biomedical Sciences and Smit Heart Institute, Cedars-Sinai Medical Center, Los Angeles, CA

[5]Department of Medicine, Jacobi Medical Center, Albert Einstein College of Medicine, Bronx, NY

[6]Department of Medicine, Massachusetts General Hospital, Harvard Medical School, Boston, MA

SUPPLEMENTAL METHODS

Glomerular Filtration Rate (GFR) measurements:

To validate estimated GFR equations in mice, we directly measured GFR, plasma creatinine and cystatin C levels in an independent cohort of mice with varying degrees of renal insufficiency (N=17). Of these 17 mice, 6 animals had ischemia-reperfusion injury (1), 4 animals had uninephrectomy, 2 animals had CKD from oxalate nephropathy (2), 1 animal had CKD from genetic ablation of podocytes (3) and 4 with no kidney disease. Plasma creatinine (Diazyme) and cystatin C (Quantikine ELISA Mouse/Rat Cystatin C Immunoassay, R&D Systems) were measured according to manufacturer's instructions. GFR was measured using a FITC-sinistrin transdermal device (MediBeacon) as described elsewhere (4). Briefly, skin fluorescence was continuously recorded by the device during 1 hour following the retro-orbital injection of 5mg/100g body weight of FITC-sinistrin. Data were extracted and analyzed using MBstudio and MBlab softwares (MediBeacon) to calculate GFR. We then tested different models (linear, polynomial, logarithmic, exponential, power) to assess the best correlation of GFR with plasma creatinine and cystatin C concentrations based on r squared values. The best correlation was found using cystatin C measurements with the linear model (Supplemental Figure 1 C-D). In all subsequent experiments, we evaluated renal function by measuring cystatin C levels by ELISA and calculating estimated GFR (eGFR) values using the following formula: GFR (mL/min/100g body weight) = -3.2886 * plasma cystatin C (μg/mL) + 2.9409.

Quantification of L-kynurenine pathway metabolites

Measurements of L-kynurenine and metabolites of L-kynurenine were performed using high performance liquid chromatography and isotope-dilutional mass spectrometry. All non-isotopic chemicals, except when noted, were purchased from Sigma-Aldrich, Saint Louis, MO; all isotopic standards, except when noted, were purchased from Toronto Research Chemicals, Toronto, Canada. Briefly, 10 microliters of plasma were mixed with 10 microliters of a 50 micromole/L mixture of isotopic standards diluted in water (L-kynurenine-d4, Quinolinic acid-$^{13}C_3$,$^{15}N_1$, Nicotinic acid riboside-d4, NAD+-d5, L-tryptophan-d5 (CDN Isotopes, Pointe-Claire, Canada), nicotinamide-$^{13}C_6$ (Sigma Aldrich, St. Louis, MO)). Plasma proteins were then precipitated by mixing with 80 µL acetonitrile (80% final conc.), and precipitates were cleared by centrifuging at 14,000 rpm for 10 minutes. Supernatant was transferred into a 96-well plate for analysis. Analysis was performed using an API 5500 triple quadrupole mass spectrometer from AB Sciex (Foster City, CA) coupled to a Shimadzu Prominence UFLC liquid chromatography system with autosampler (Shimadzu Scientific Instruments, Colombia, MD). High performance reverse phase chromatography was performed by injecting 15 microliters of plasma supernatant onto a Kinetex Polar C18 UPLC column (150 x 4.6 mm, 2.6 µm bead diameter, 100A pore size, Phenomenex, Inc., Torrance, CA) using a gradient elution protocol detailed in Supplemental Table S2. MS/MS transitions and settings for multiple reaction monitoring (MRM) of each metabolite is shown in Supplemental Table S3. Instrument control, data acquisition and quantification were performed using Analyst 1.6.2 software (Sciex, Framingham, MA). Calibration standards for each metabolite (L-tryptophan, L-kynurenine, quinolinic acid, kynurenic acid, nicotinamide, nicotinic acid,

NAD+, nicotinamide mononucleotide, and indoxyl sulfate) were diluted into phosphate buffered saline, standard curves for each metabolite were generated by running calibrator mixtures at 0, 12.5, 25, 50, and 100 micromoles/L for each metabolite. L-kynurenine-d4 was used as the internal standard for kynurenic acid and nicotinamide mononucleotide, quinolinic acid was used as internal standard for indoxyl sulfate. NAD+-d5 was internal standard for NAD+ and NADH measurements. Metabolite concentrations were measured by quantifying the area under the curve (AUC) for each MRM transition and normalizing it to its respective isotopic standard and metabolite/internal standard ratio was converted into absolute concentrations according to each standard curve.

SUPPLEMENTAL REFERENCES

1. Poyan Mehr A, Tran MT, Ralto KM, Leaf DE, Washco V, Messmer J, et al. De novo NAD(+) biosynthetic impairment in acute kidney injury in humans. *Nat Med.* 2018;24(9):1351-9.
2. Luz HL, Reichel M, Unwin RJ, Mutig K, Najenson AC, Tonner LM, et al. P2X7 Receptor Stimulation Is Not Required for Oxalate Crystal-Induced Kidney Injury. *Sci Rep.* 2019;9(1):20086.
3. Stotter BR, Talbot BE, Capen DE, Artelt N, Zeng J, Matsumoto Y, et al. Cosmc-dependent mucin-type O-linked glycosylation is essential for podocyte function. *Am J Physiol Renal Physiol.* 2020;318(2):F518-F30.
4. Scarfe L, Schock-Kusch D, Ressel L, Friedemann J, Shulhevich Y, Murray P, et al. Transdermal Measurement of Glomerular Filtration Rate in Mice. *J Vis Exp.* 2018(140).

SUPPLEMENTARY TABLES

Table S1: Raw data for Metabolomic profiling in non-pregnant state and during pregnancy in uninephrectomized mice and controls

Table S2. HPLC setting for Reverse Phase Chromatography

Table S3: MRM Mass Spectrometer Settings for isotope dilutional quantification of metabolites

TABLE S1

Label	Sham								UNx							
	NP				GD18				NP				GD18			
Mevalonolactone	0.03592652	0.46972178	0.21120939	0.09239365	0.18409199	0.17564547	0.04203073	0.08545017	0.89535883	0.31797375	0.09871632	0.11852656	0.15863056	0.02970752	0.03518536	0.03986602
2-2-Dimethyl Succinic acid	77.152	74.98716	44.43343	62.95543	80.06378	92.28647	107.58639	61.28098	264.34096	66.20572	71.80722	45.88379	32.28491	39.21382	43.66312	39.95206
2-3-Dihydroxybenzoic acid	0.01353847	0.04652243	0.02028266	0.07201167	0.02630498	0.06189978	0.05231303	0.02980433	0.04614761	0.01364109	0.09198886	0.01360292	0.01037927	0.03130797	0.01303699	0.04186383
2-3-Dihydroxyisovalerate	0.2014902	0.06994288	0.07095504	0.07918642	0.44682169	0.17433128	1.26135811	0.04209452	1.72039545	0.08457284	0.04460085	0.38784598	0.48587981	0.05053254	0.10543489	0.10397233
2-3-Pyridinedicarboxylic acid	12.88607	18.355	17.43726	17.95731	27.21121	20.23695	55.60807	42.27985	35.15602	12.18171	16.42523	11.15114	10.24268	17.08951	19.17495	25.4601
2-4-Quinolinediol	0.081817518	0.035490123	0.038503335	0.014707506	0.455546042	0.081692922	0.180682092	0.131311197	0.110387695	0.031706689	0.019347486	0.008721645	0.018745098	0.076195501	0.019380257	0.246495632
2-Deoxyadenosine 5-diphosphate	0.035348059	0.006061512	0.008429529	0.00959796	0.025571812	0.117897749	0.063440364	0.018429186	0.003609168	0.085270978	0.004198438	0.002213024	0.003524559	0.008645027	0.01330023	0.049841309
2-Deoxyadenosine 5-monophosphate	0.10568521	0.06198275	0.14676905	0.01700596	0.02376243	0.02201736	0.05216522	0.02588204	0.05082746	0.04809134	0.029291	0.01074664	0.01494893	0.01341695	0.02835528	0.06203326
2-Deoxyadenosine	1.7982268	0.4160302	17.1822185	0.2613078	0.53335	0.4240913	1.7065713	0.6062236	1.2843097	5.0313018	1.6913008	7.8769425	0.3667721	1.0296688	1.9170904	44.5933796
2-Deoxycytidine 5-diphosphate	10.1784566	8.2139877	2.9447209	1.4254374	2.1475641	13.8519636	4.5501386	10.5975959	0.150928	3.8937881	2.8040882	0.562218	0.9978934	0.7652748	1.41562	0.8102191
2-Deoxycytidine 5-monophosphate	0.008339119	0.134652865	0.022474642	0.06068424	0.081152307	0.036514298	0.040723888	0.024027272	0.080416613	0.077558917	0.00884004	0.01136807	0.016108339	0.010710788	0.008253328	0.020997341
2-Deoxycytidine	29.95558	44.55831	44.26609	15.90066	57.24152	36.70761	41.3232	61.03696	18.96646	41.90204	45.30802	22.20892	16.89542	17.643	16.93361	17.35687
2-Deoxy-D-glucose 6-phosphate	1.1090637	1.1671982	0.5156401	0.5884713	7.6565396	2.7503806	1.0430786	1	0.371619	0.7889712	1.3979397	0.9850643	0.3186589	0.7596792	1.2080437	1.4945682
2-Deoxy-D-ribose	58.923443	1.983782	6.347189	74.069733	3.786372	40.014776	5.108894	5.870629	3.189944	1.884876	13.455662	8.107746	4.636873	36.892431	2.103516	6.510297
2-Deoxyguanosine 5-diphosphate	0.81445859	0.25574052	0.10692298	1	0.05361781	0.57454037	0.13661217	0.47464509	0.24961895	0.15679949	0.39004921	0.1865029	0.20257726	0.817486	0.38250052	0.19015638
2-Deoxyguanosine 5-monophosphate	36.07343281	25.40303595	3.70690381	53.22647243	0.70443212	1.11425304	0.19286407	16.73927377	5.04997016	2.27519108	2.78842028	24.73049439	14.47346419	1	0.65205673	0.04928103
2-Deoxyguanosine	0.003655798	0.007914847	0.026718251	0.009792369	0.045080404	0.050036305	0.037738335	0.047103014	0.108003127	0.010120012	0.015765507	0.004552287	0.003778133	0.00530462	0.003122139	0.045739981
2-Deoxyinosine	0.027354624	0.04034576	0.204324512	0.249804908	0.042927616	0.096765644	0.028246197	0.021809371	0.144923153	0.065987643	0.020729982	0.757435642	0.088629612	0.012320454	0.005068023	0.050435311
2-Deoxyribose 5-phosphate	19.8539483	5.5453355	1.5276549	18.5630137	6.0727238	12.5437542	1.4409654	1.102893	0.8580336	3.0497693	4.263698	0.8776886	3.6628382	5.0173431	4.3199671	4.6482751
2-Deoxyuridine 5-triphosphate	0.115698871	0.01583569	0.015736596	0.020041362	0.075807591	0.023137869	0.048804145	0.029302206	0.053069322	0.019240655	0.088382502	0.011341799	0.014365167	0.00968276	0.012068355	0.003589082
2-Deoxyuridine	44.28561	77.51572	47.106025	38.283707	36.602467	103.189002	117.608508	46.787545	75.491768	6.691374	37.38849	24.307036	21.739208	34.033342	38.845897	34.675551
2-Isopropylmalic acid	205.004527	114.630899	7.455325	84.800996	179.176745	129.145012	467.540062	145.495019	216.177597	105.238328	8.595306	95.706186	7.101653	5.372842	132.161898	226.455619
2-Ketobutyrate	7.994153	6.346976	30.211477	10.616019	64.283378	6.875881	4.761583	8.275278	35.919248	4.144003	43.487959	17.668361	4.75646	2.492895	3.234439	33.965579
2-Methyl-1-butanol	24.089612	22.529224	17.004137	16.472254	38.913608	20.906397	64.079758	35.892671	30.65326	24.074032	16.70139	4.037888	10.5466	16.96138	20.428221	50.091454
2-Phosphoglyceric acid	1.8068705	8.3965637	6.8509504	6.3260836	7.3047123	4.7115199	7.1768308	6.6604626	10.8486161	0.2238554	5.1385088	3.7947292	0.2716857	1.304169	3.3373722	7.9522842
3-2-Hydroxyethylindole	0.066365402	0.078117288	0.142084574	0.006502983	0.110382987	0.028127549	0.077423257	0.021681578	0.021240166	0.20620155	0.152271527	0.067440769	0.111168324	0.011984973	0.020859854	0.028781452
3-Dehydroshikimic acid	0.167237175	0.039559809	0.007115132	0.063382879	0.056977893	0.068098153	0.077970194	0.068623684	0.338125892	0.072112161	0.044738236	0.007636206	0.091805622	0.006213642	0.003450292	0.052669218
3-Hydroxyanthranilic acid	0.020622473	0.016024671	0.022216211	0.007819757	0.144363272	0.025246344	0.059329545	0.070244467	0.046756906	0.028629246	0.029237078	0.015032451	0.044561176	0.015172533	0.074841808	0.010027632
3-Hydroxy-DL-kynurenine	0.029392113	0.00642045	0.040497722	0.015097667	0.27040094	0.011810441	0.216106537	0.540736509	0.026761931	0.093509276	0.154535032	0.026375026	0.160493693	0.10533308	0.009787135	0.081099815
3-Hydroxyphenylacetic acid	0.013068402	0.028590774	0.042376177	0.026023218	0.083101678	0.020087818	0.024770418	0.001789741	0.034203758	0.034634157	0.035228922	0.02845081	0.024505618	0.018067192	0.023520734	0.057941833
3-Indoleacetic acid	28.60004	22.60095	32.2021	41.90013	50.09207	26.76605	57.56469	27.06677	87.00413	43.41495	53.98029	21.99271	27.89867	29.79613	25.50069	81.32786
3-Methylglutaric acid	73.60625	71.55039	81.01456	62.96215	76.73768	80.92222	88.46783	74.48832	429.36674	40.4587	49.22778	43.88606	23.0301	40.0775	477.49644	39.93459
4-Aminobenzoic acid	0.068610138	0.018988285	0.009821444	0.032044147	0.040051142	0.01124643	0.041682296	0.028141243	0.061187199	0.025290741	0.008373014	0.01140844	0.017976109	0.007621273	0.051351169	0.00946066
4-Guanidobutyric acid	0.038082393	0.018505952	0.00645413	0.021791696	0.120284629	0.039389621	0.119682523	0.047714267	0.002519555	0.066999169	0.020562546	0.035158688	0.056061176	0.02637858	0.055930611	0.031183045
4-Hydroxybenzoic acid	1.84356057	0.41065575	2.47131988	2.32245186	0.1516695	1.81899744	3.45335253	0.06437094	5.6918744	3.0275307	6.09712043	0.0728762	1.53392587	0.18892021	1.6145032	26.20250132
4-Hydroxy-L-glutamic acid	0.02127224	0.03046638	0.06310946	0.03305862	0.01384216	0.02150901	0.0408748	0.02289878	0.06134926	0.01603046	0.02983495	0.00892137	0.0287368	0.03839164	0.01245418	0.02501162
4-Hydroxyphenyl-pyruvic acid	0.6061001	5.7162576	9.6898437	3.1232506	2.9422078	5.5651571	1.7297406	7.7584639	2.7712269	8.24978	20.835235	0.6291019	7.1815169	8.1750856	0.4002937	32.8730864
4-Methyl-2-oxovaleric acid	26.463749	1.961132	40.762061	2.109951	42.03356	13.074273	43.304427	10.29693	41.475051	2.886929	20.475902	24.424745	16.812645	1.291792	2.336601	1.973781
4-Pyridoxic acid	22.562322	6.759376	16.903098	28.741655	80.34194	41.782348	86.904698	86.951766	79.87191	36.736039	58.121434	14.055614	48.198512	34.741075	54.618254	89.871356
4-Quinolinol	0.009215971	0.045365437	0.032520827	0.063655157	0.022942863	0.041337391	0.011259554	0.100912321	0.018920633	0.023453218	0.006272478	0.022238763	0.012235382	0.038013364	0.009501711	0.057771305
5-Deoxy-5-(methylthio)adenosine	4.0181864	0.6213622	1.6306327	3.6660351	10.0753164	5.3932032	11.1554778	12.9364081	16.410802	4.2822822	9.4399021	3.2157782	10.203781	5.9228591	5.8499457	11.0715147
5-Hydroxy-3-indoleacetic acid	0.13525541	0.07649005	0.04630144	0.01818545	0.23191271	0.13127704	0.1278022	0.17914607	0.11671544	0.25329403	0.08877827	0.08142599	2.71092043	0.25042498	0.13681722	0.4195082
5-Methoxytryptamine	0.020981978	0.012348206	0.022546829	0.001655692	0.023559548	0.096582844	0.01418736	0.008677087	0.033747772	0.013087601	0.00305184	0.008893412	0.008780348	0.037581841	0.015735176	0.031026848
6-Hydroxynicotinic acid	0.0322971	0.3533134	0.3631383	0.07470103	0.31249665	0.11786379	0.11609615	0.73977604	0.16493946	0.06626435	0.03420212	0.13884475	0.08771979	0.02739201	0.1066923	0.07275724
Adenine	1.12302714	0.77264746	0.80226508	0.80552946	1.35629444	0.46640506	0.24813352	0.04841255	0.14093011	1.82964983	1.81082055	1	0.53394647	0.44411887	0.47261598	0.74996969
Adenosine 3-5-cyclic monophosphate	21.90885127	9.21313179	19.7469105	19.63824337	0.72205959	10.77282869	0.01879778	6.29482259	54.25348816	9.53070703	4.02957785	12.98720006	8.36321684	5.29667244	5.51566354	0.01433933
Adenosine 5-diphosphate	0.44641349	0.20946098	0.19732202	0.38660005	0.18090087	0.24837328	0.54384957	0.38090953	0.22926432	0.07421115	0.16022813	0.04828988	0.34751519	0.01462356	0.04647854	0.12909529
Adenosine 5-monophosphate	51.28912085	26.00689156	22.38263152	50.71360576	0.04111103	10.00333944	0.04507391	15.3752205	1.27480902	5.79225567	26.72354853	20.01405277	55.3905272	39.42191528	8.06770025	0.05989799
Adenosine 5-triphosphate	0.027054864	0.037438085	0.023594934	0.011846767	0.010307045	0.131193743	0.025154578	0.027023056	0.365007858	0.010613804	0.02318763	0.014761165	0.012807952	0.004738765	0.012016075	0.008045175
Adenosine	0.02262913	2.00369248	0.07005273	0.82577993	0.60030088	0.69951398	2.24013108	1.26634246	0.06291076	0.06613893	0.03481454	0.67831959	0.39743182	0.23760386	0.59757382	1.16388672
Adenylosuccinic acid	0.002662825	0.015671691	0.008951485	0.12961368	0.021281427	0.011519541	0.05576505	0.069561219	0.006459205	0.001031086	0.032429507	0.001388362	0.011814314	0.018251357	0.00643658	0.00124721
Adipic acid	74.16964	68.59711	74.12595	30.60321	72.00005	84.34712	91.22007	61.92441	87.43779	75.48697	61.70809	45.16416	33.32737	39.83151	39.89983	56.08635
AICAR	0.11401889	0.21351418	0.06673374	0.60115865	0.30471225	0.10933181	0.31916142	0.27615543	0.06151208	0.17482694	0.02759976	0.07287607	0.37659082	0.12353996	0.11750468	0.3642098
Allantoin	15.033945	15.876709	18.951188	13.150752	53.517003	120.051447	46.324551	24.716831	32.242648	33.459351	37.870858	7.660401	11.528169	16.820422	22.625819	25.964282
alpha-D(+)Mannose 1-phosphate	12.499049	5.954079	8.440422	1.007665	44.986189	4.001648	3.691264	17.517258	1.495901	2.399136	1.967228	3.527739	12.441738	10.434549	7.685341	12.382161
alpha-D-Glucose-1-phosphate	4.2312253	2.2632084	3.8781874	0.1362808	27.4277559	3.2595758	9.4590007	0.24712	0.5637651	0.3008913	2.1269769	1.4781034	1.4602684	14.8079689	1.1176725	13.8782356
alpha-Ketoglutaric acid	800.6745	338.0861	1051.2454	816.4054	1923.6989	603.6995	2412.9545	1683.0833	2789.9636	1328.1394	2257.144	656.5632	854.1284	820.3371	1623.5157	1336.2209

**Arabinose-5-phosphate**	379.325082	153.0276728	494.840509	2.70441812	1.5567903	0.44729452	0.33117743	0.92095854	0.41681714	0.08902867	0.2992766	210.1303824	1.10880575	30.06254314	0.61713992	1.16885127
**Argininosuccinic acid**	0.85989408	0.57872769	0.46451867	0.14798329	0.33206533	0.25575466	0.19439953	1.85144971	0.58117619	0.12210319	0.43786182	1.05226285	0.04680283	0.15223103	0.05611215	0.2094736
**beta-Nicotinamide adenine dinucleotide**	0.016110996	0.037581832	0.023546302	0.025301606	0.015466462	0.038455551	0.063203843	0.017651903	0.014546317	0.008515386	0.006085965	0.017066537	0.002958581	0.016433945	0.094996046	0.058825462
**beta-Nicotinamide mononucleotide**	0.018667236	0.013056168	0.036294816	0.030056698	0.010945822	0.037596885	0.015002544	0.06364478	0.093405828	0.073251123	0.009713118	0.115373837	0.056635441	0.011959387	0.006016911	0.022068637
**Cellobiose**	3.8334145	2.6800192	2.6889046	3.0765538	0.6568032	3.0730505	3.4007616	0.6781089	3.6238688	2.4385131	3.1613959	1.3449281	1.1303334	1.0497104	1.4286985	1.8272343
**Chorismic acid**	28.7466861	0.8188743	15.8805392	12.9114401	22.2953282	23.4749648	47.4663108	15.2599849	3.616477	25.3132343	25.9306941	9.2606649	0.8175329	15.9567308	19.3058794	16.5837143
**cis-Aconitic acid**	473.1549	666.5593	1444.7669	1037.7929	2923.0957	764.5947	2166.4398	755.0382	4274.9832	1610.2165	1190.635	886.2033	1650.0039	871.8382	935.5976	1055.0053
**Citramalic acid**	44.13338	31.022	52.0118	65.91792	67.02916	55.07577	106.9823	51.5878	96.60821	60.77126	62.34911	29.78006	38.8755	40.41843	52.85079	68.48343
**Citric acid**	10803.102	9097.04	9643.56	12484.496	16285.198	11686.489	27111.001	17708.369	34181.346	11688.953	15029.359	6595.292	10973.996	13841.1	14468.925	8376.514
**Creatine phosphate**	0.07596837	0.0163661	0.04336289	0.0329312	0.03719868	0.13251502	0.27780913	0.07020392	0.04757785	0.34607398	0.03464195	0.01480237	0.06428427	0.02719212	0.0486907	0.1709904
**Creatine**	16.504729	17.844396	21.32291	13.005296	12.275349	16.285092	9.867221	19.200999	36.713036	14.094858	15.042225	13.871161	5.325032	11.840868	3.358657	5.183483
**Cytidine 5-diphosphate**	0.004238893	0.003898101	0.044245263	0.01847435	0.091439677	0.040754621	0.02853316	0.073240423	0.039562614	0.003072259	0.02898476	0.035520411	0.005952803	0.001777024	0.017085784	0.034725327
**Cytidine 5-triphosphate**	0.10809619	0.035518968	0.213023002	0.071280397	0.040153086	0.03544553	0.022791406	0.026830102	0.038324452	0.044895509	0.023787931	0.034454167	0.010877883	0.022829015	0.006631153	0.012265772
**Cytidine**	5.5028324	4.3235732	9.4921874	3.2614239	3.9246076	7.0979605	10.4521087	1.6662281	0.9121008	4.4699659	3.6778449	6.4336181	0.7919012	1.2503116	5.6376635	7.1512273
**Cytidine-5-monophosphate**	0.01346555	0.03680729	0.0533991	0.14242313	0.0820973	0.0296805	0.20577822	0.17560748	0.05045224	0.09829262	0.09564586	0.01019108	0.02363738	0.05512708	0.09504877	0.01685955
**Cytosine**	0.038442839	0.001962916	0.004150916	0.045028312	0.0041319	0.124830564	0.004306619	0.046622248	0.006021745	0.011058648	0.029553659	0.018300079	0.000808924	0.001083579	0.016825735	0.011741532
**D-+-Galactosamine**	0.18883141	0.07945594	0.63223752	0.44441517	0.61410826	0.1454211	0.12426776	0.07922255	0.08231198	0.54976286	0.65407449	0.02725468	0.50323086	0.17541923	0.23137741	0.36841829
**Deoxyadenosine 5-triphosphate**	0.035278498	0.009067743	0.027959046	0.042699042	0.004080508	0.069712317	0.049894113	0.007472219	0.018615722	0.020330404	0.009474393	0.004779407	0.013614873	0.002579211	0.00503296	0.009279265
**Deoxycytidine 5-triphosphate**	0.54956722	0.23711832	0.37920427	0.27132594	0.27941603	0.60380883	0.32643316	0.15848611	0.08741331	0.5405743	0.30016725	0.06741787	0.06374939	0.02203371	0.2995275	0.34926892
**Deoxyguanosine 5-triphosphate**	0.014973993	0.014843839	0.018455339	0.009442333	0.028365405	0.144705405	0.364921328	0.036304805	0.039121053	0.023587377	0.020071852	0.011790436	0.005740126	0.030373749	0.014627294	0.024379068
**Deoxythymidine 5-triphosphate**	0.020460346	0.013519665	0.014173352	0.015118417	0.014018732	0.011794464	0.015827208	0.020447684	0.032124357	0.011119932	0.023713152	0.014228715	0.009914239	0.009959945	0.006116156	0.029152135
**D-erythro-Dihydrosphingosine**	0.16812576	0.13866622	0.11395917	0.04328966	1.28421572	0.87590385	7.44932062	0.08110797	0.23431307	0.15395066	0.10376519	0.12926938	0.13167065	1.25671035	1	2.54322809
**D-Fructose 1,6-biphosphate**	1.207397884	1.474264885	0.430872774	0.364204183	0.300235899	0.786032278	0.009558554	2.545122764	1.21072606	0.544249062	0.698655337	1.40336806	0.507215042	0.239440626	0.678451179	0.246691508
**D-Fructose 6-phosphate**	2.98275	7.53899	14.57473	17.463838	133.444607	4.779847	56.037978	31.309629	1.685165	3.717815	4.473879	5.378186	15.984083	31.149924	2.928837	6.334807
**D-Gluconic acid**	15.15462	143.08718	197.73575	353.50331	58.42497	240.49827	580.44783	301.7866	108.21827	207.25528	338.13451	133.43096	239.17182	237.01595	315.78183	629.18006
**D-Glucosamine 6-phosphate**	0.03845465	0.03126827	0.02191515	0.01589604	0.49520317	0.09482565	0.08738432	0.03951048	0.05201354	0.04806811	0.02166401	0.04813707	0.0201992	0.08709091	0.0337889	0.04371013
**D-Glucose 6-phosphate**	2.98275	7.53899	14.57473	17.463838	133.444607	4.779847	56.037978	31.309629	1.685165	3.717815	4.473879	5.378186	15.984083	31.149924	2.928837	6.334807
**Dihydroxyacetone phosphate**	0.27726264	10.25540097	4.63425263	0.05889198	0.64807178	3.6092006	0.56352959	2.3638444	0.42396776	2.18380394	0.04583151	0.10340029	0.52049224	1.10905366	0.44728368	0.41590131
**DL-2-Aminoadipic acid**	14.819475	18.446955	14.723767	19.828536	23.322811	21.333077	2.206383	24.713195	26.995705	15.375212	19.936384	16.76079	11.924882	16.221185	13.014605	24.106047
**DL-Glyceraldehyde 3-phosphate**	0.1325208	1.3614115	0.327904	5.7545654	0.5633874	1.3788408	0.3138523	1.1766309	5.7409397	1.5450457	3.4725648	5.7020933	0.9154326	0.3476453	1.2729662	1.6381194
**DL-Isocitric acid**	11112.344	9419.694	9983.984	12830.435	16784.146	11999.685	27964.299	18243.372	35404.757	12087.238	15466.183	6831.202	11294.779	14404.729	14813.738	8236.997
**D-Maltose**	2.2834791	1.857309	2.6645471	5.5046492	0.9781519	3.3137344	6.1096315	2.6487177	4.5708397	3.2719559	3.6680122	1.4286199	1	1.0483385	1.7547665	2.2791449
**D-Mannose**	1426.7699	1072.9037	1087.4146	1006.7919	902.0586	951.7217	1624.1049	993.7725	1787.401	1492.0967	1170.107	783.6796	404.0634	560.0692	679.1959	994.5562
**D-pantothenic acid**	316.84417	344.61686	313.00904	274.89069	79.87811	69.70365	99.09513	107.8266	569.75633	308.29818	296.20498	168.68078	38.07337	42.80533	35.86252	115.65139
**D-Ribose 5-phosphate**	527.5667261	215.4675251	687.3047646	1.9187324	0.2943656	20.75671	0.6577691	1.010515	2.3694799	1.4430438	0.5962767	294.0398562	0.579962	2.0319995	1.703106	1.2135174
**D-Ribulose 1,5-biphosphate**	0.34104007	0.71459341	0.73963512	0.40267346	0.40774006	0.20049318	0.52803006	0.06899279	0.3870159	0.63542598	0.53943354	0.14451055	0.37167346	0.19431551	0.01776581	0.28641081
**D-Sedoheptulose-7-phosphate**	0.53612327	0.12929789	0.16728406	0.45927109	11.10565038	0.76507241	28.32740733	4.24654828	0.33983532	0.21366422	0.07451718	1.33647639	3.15672283	3.01163324	0.60038791	25.90836659
**D-Xylose**	11.064658	20.846865	20.772706	11.374721	18.638464	12.766091	34.575152	7.66396	22.974196	19.358156	51.594081	10.28291	5.096677	5.533815	10.276864	12.104527
**D-Xylulose-5-phosphate**	2674.149609	1075.044728	3517.421851	11.499534	24.684898	8.421728	30.845628	10.020393	12.561425	6.383829	6.928535	1521.423191	290.511912	15.823682	14.088419	19.465493
**Epicatechin**	0.013314968	0.024484633	0.011196999	0.065612826	0.086576419	0.021124171	0.027883848	0.139627091	0.062552314	0.036576446	0.018747616	0.007543591	0.002470324	0.009166506	0.022597509	0.018968434
**Flavin adenine dinucleotide**	0.14861831	0.06039585	0.08459481	0.1098639	0.0416693	0.13618337	0.06917693	0.08492965	0.51654002	0.09682357	0.09793756	0.01526407	0.08444928	0.03973619	0.35009255	0.05089195
**Folinic acid**	0.005352834	0.00636401	0.030538469	0.015882473	0.182160503	0.045855287	0.004989094	0.024370026	0.007482065	0.000534326	0.028944013	0.002470945	0.014740053	0.007487395	0.001791583	0.024731798
**Galactonic acid**	15.15462	143.08718	197.73575	353.50331	58.42497	240.49827	580.44783	301.7866	108.21827	207.25528	338.13451	133.43096	239.17182	237.01595	315.78183	629.18006
**gamma-Aminobutyric acid**	0.004164352	0.012577265	0.076184519	0.020665013	0.012980504	0.060099415	0.025234225	0.047172235	0.003911522	0.049069454	0.017529777	0.037858806	0.014058278	0.028887287	0.019980214	0.072790758
**gamma-Glu-Cys**	0.012582059	0.043997646	0.055491257	0.009109822	0.02310904	0.02528218	0.08375562	0.01966042	0.027650729	0.106742418	0.005925425	0.025012102	0.013508802	0.03707495	0.016965367	0.021462885
**Glucoheptonic acid**	0.36705793	0.72102698	1.10216018	0.34264764	0.06247355	1.04229935	0.14424979	0.31018934	0.06313154	1.08763555	0.02934838	0.07129072	0.28793383	0.20275798	0.47277829	0.21785831
**Glyceric acid**	92.2553	68.58134	97.07339	112.18996	99.95255	106.81077	227.74428	135.68933	91.38239	74.4712	75.2587	52.98644	48.92623	66.51901	93.27469	147.63986
**Glyoxylic acid**	0.37683545	0.0855378	0.56466444	0.0759077	0.04944695	0.63459016	0.33438543	0.10976942	0.60566989	0.07068192	0.20384891	0.06693745	0.07807645	0.01729222	0.06732323	0.07434376
**Guanine**	0.024481099	0.006467668	0.023902939	0.005865345	0.00205762	0.058743986	0.046949149	0.074893339	0.034111057	0.011398879	0.03283816	0.003406428	0.019466962	0.018639612	0.03668861	0.03410982
**Guanosine 3,5-cyclic monophosphate**	1	0.1457544	0.31391986	0.08681461	0.02401815	0.16770667	0.04626818	0.03158078	3.36771638	2.04924068	1	0.43872605	0.08245726	0.73610857	0.21208433	0.12536987
**Guanosine 5-diphosphate**	0.018083791	0.064856182	0.037838139	0.015347522	0.037075314	0.047647929	0.024088961	0.067518085	0.030177408	0.021252498	0.013050796	0.036704527	0.010712404	0.012373385	0.001469968	0.001737193
**Guanosine 5-triphosphate**	0.007976412	0.090132958	0.028760334	0.047554844	0.01235007	0.00875097	0.017373609	0.048159136	0.013596115	0.07806354	0.021394324	0.002124182	0.004875257	0.018053941	0.017689349	0.020274884
**Guanosine**	0.049112432	0.040955697	0.415040069	0.028676022	0.04875708	0.074759238	0.213809146	0.04053078	0.023158225	0.155159181	0.038250874	8.831181404	0.115278325	0.017498695	0.005057465	0.031633038
**Homocitrate**	5.907299	13.866399	8.765831	16.190197	14.062423	12.592815	28.238533	25.297925	79.780523	3.321831	23.257381	6.519075	30.669662	11.104326	14.975043	13.062825
**Hypoxanthine**	0.04043282	0.11842276	15.85830493	0.04177593	0.03210403	0.19628158	0.10816674	0.02675032	0.14171794	0.04128854	0.01382383	5.43657751	4.18206468	0.02854533	0.01430673	0.08948667
**Indoline-2-carboxylate**	0.14456767	0.27302511	0.08069381	0.1173131	0.06827625	0.15055509	0.11489641	0.06450285	0.40550542	0.11486139	0.05637861	0.06400824	0.08004374	0.15376567	0.37019781	0.17012659
**Inosine 5-diphosphate**	0.2216312	0.7793208	0.18209202	0.16373904	0.45413963	0.17237966	0.59070814	0.23166492	0.06286129	0.20630182	0.24943518	0.21645301	0.01747748	0.07286621	0.09508189	0.34036631
**Inosine 5-monophosphate**	0.46550394	0.29459282	0.53999921	3.79262117	0.07750788	0.20520473	0.04127835	0.10425878	0.07295708	0.25638931	0.16608464	5.13592249	0.41235417	1.15991085	0.07328472	0.07382415
**Inosine 5-triphosphate**	0.034372241	0.026595436	0.013739721	0.025690393	0.01828341	0.026813927	0.058647641	0.041695508	0.044834888	0.084709822	0.007585336	0.005967911	0.010884554	0.00913825	0.03259679	0.03066056

**Inosine**	0.98209894	0.94401958	791.3262762	0.23957395	1	0.45167311	0.73537546	0.59688407	0.5522516	0.09882283	0.09962561	105.6748503	41.28307391	0.26031331	0.3849429	0.10919147
**Isopentenyl pyrophosphate**	0.029301834	0.23337176	0.200204124	0.015961688	0.021626967	0.037665245	0.139102702	0.010350538	0.36586532	0.138467369	0.074088834	0.017801802	0.013678967	0.04496324	0.007683706	0.08144349
**Isopentyl acetate**	212.3901	182.2611	188.0234	234.9062	251.6241	214.8291	379.3396	267.3114	631.674	166.6689	240.7909	125.0118	150.6799	183.5529	181.7868	257.442
**Itaconic acid**	18.788762	3.257698	12.569365	7.523199	39.627816	16.38251	36.958371	1.342964	25.03646	2.645245	14.83709	4.369982	2.542026	11.859359	2.633075	26.050951
**Ketoisovaleric acid**	2.7586563	1.6704074	1.9610173	3.9601544	29.8836378	59.2025556	75.5209099	40.3598907	1.2331297	2.3734631	0.7559267	2.0923129	0.6041192	0.5952641	1.6451719	36.1609674
**Ketovaleric acid**	55.90087	73.15374	115.50551	116.77473	245.08254	143.33193	418.39016	181.8472	44.59452	153.34448	242.08745	70.12435	96.33387	117.87132	160.38376	308.93606
**Lactic acid**	6268.766	6371.41	6439.968	5407.046	8299.48	7034.274	11093.331	6388.499	7584.982	6661.053	5328.934	4084.887	3573.43	3298.516	3600.232	6208.799
**L-Arabinose**	7.2986557	2.4917718	17.4572723	0.6295907	6.4947241	5.6618322	10.7046377	3.0083716	2.1533629	7.9312232	2.5367199	0.6156851	5.8945627	1.9190626	1.3039364	0.4630451
**L-Arabitol**	0.7550279	0.7011073	1	0.579145	2.9027056	1.931519	1.5618463	1.0985581	1.4415492	1.5022336	2.6543975	0.7832834	1.7294003	1.9265503	2.0441087	0.5535601
**L-Arginine**	0.33141	1.8107163	0.2691726	1.2844979	1.1690884	0.2535428	0.3872656	0.4815538	0.7407263	0.650978	0.2158577	0.3176121	0.1752709	0.1058627	0.245709	0.5583308
**L-asparagine**	3.0264588	3.4400175	4.4879658	3.0170579	0.3972448	5.8419965	5.3917522	3.3533922	4.0549351	4.9860309	2.63632	3.4919964	1.7653716	1.516936	2.3186109	4.975887
**L-Aspartic Acid**	790.1079	1277.7928	1074.4902	962.7881	430.6663	526.6605	410.6977	331.5895	1657.7845	593.5947	104.3611	349.4124	413.8995	324.8914	143.9886	115.9255
**L-Canavanine**	0.040093638	0.029126309	0.018396084	0.018993739	0.0314522	0.086572629	0.027892468	0.043293751	0.004020927	0.020939513	0.033942126	0.02749714	0.001662152	0.005163541	0.000978672	0.001309443
**L-Carnitine**	1.567176	1	1.2682859	1.5538575	4.2575105	2.6326162	3.0956802	1.9853062	3.6373759	1.2304605	1.2483439	0.7880846	1.3218597	0.404031	0.9981829	1.1734588
**L-Citrulline**	13.754071	18.590492	14.557887	14.575552	13.493169	17.902409	39.05532	10.170099	28.233747	28.125216	27.260365	30.417326	16.296508	17.984867	9.432563	16.164634
**L-Cystathionine**	0.109142466	0.112080525	0.112838184	0.083131393	0.172042228	0.072125562	0.107126581	0.083216845	0.190330488	0.023810919	0.225112105	0.00853235	0.002823623	0.013088773	0.045623897	0.066448354
**L-Cystine**	0.068460278	0.135959927	0.081058726	0.032974621	0.033162244	0.30690678	0.178687348	0.020664269	0.643489021	0.07579566	0.081127461	0.188783032	0.009825222	0.023489217	0.087119971	0.024943388
**L-Dihydroorotic acid**	0.42327419	1.11680208	0.17112661	0.08613685	1.41128122	0.29062405	2.96673454	3.4731277	0.209385	3.74583444	1.89535375	2.2063753	0.7343893	0.13758481	1.30477273	1.24837055
**L-Glutamic acid**	547.839	627.9807	720.3663	716.8883	876.0506	834.7299	1114.727	826.7145	1235.2087	657.3956	677.4856	395.8915	739.9954	834.3813	470.5972	752.5054
**L-Glutamine**	28.10433	26.28922	21.09187	30.26597	34.59069	21.09461	84.33426	32.22681	49.43121	28.56746	21.02355	14.70347	24.57398	20.59301	25.48085	22.74708
**L-Gluthathione (oxidized)**	0.049912198	0.051663591	0.003577789	0.00187758	0.047709767	0.035239957	0.065285619	0.013332346	0.26511975	0.015452096	0.066591479	0.052188589	0.029933251	0.013112434	0.043791832	0.04903314
**L-Histidine**	3.838505	3.874197	4.297664	4.629482	2.044944	3.528978	2.889541	2.445578	5.208148	2.840715	4.797634	1.77487	1.645715	1.05326	1.428551	4.962503
**L-Homocysteine**	0.004815511	0.002517908	0.005313289	0.001061941	0.0534252	0.037198773	0.111289346	0.039500321	0.03587236	0.003298806	0.00424746	0.03231068	0.001212204	0.0016823	0.01993171	0.001712323
**L-Homocystine**	0.03555467	0.13614179	0.05199115	0.02819287	0.01682423	0.06457818	0.03054337	0.06556521	0.02261998	0.02354262	0.02730744	0.08489396	2.20893321	0.02179954	0.02726536	0.04099069
**L-Homoserine**	0.002748845	0.143260526	0.049145007	0.081261796	0.055689361	0.015753673	0.029949692	0.133650846	0.014068546	0.002955184	0.001767799	0.006662701	0.046656267	0.027825164	0.023883529	0.011513826
**L-Hydroxyglutaric acid**	200.315	166.7906	473.297	340.4801	430.7293	257.7956	510.0459	318.4211	713.1902	384.6928	396.0541	146.6006	234.7401	250.4098	343.6763	349.1652
**Lipoamide**	7.340379	7.2458	10.561786	9.874272	13.065161	6.028137	13.304204	12.816682	35.67595	11.37688	17.609022	5.743908	9.759847	10.163884	11.327096	22.825336
**L-Isoleucine**	4.52495	6.824382	6.156799	11.008135	8.319774	13.602243	11.834081	11.624665	25.364987	11.129851	5.706917	6.225185	1.994717	1.72032	3.896821	7.780639
**L-Kynurenine**	0.9944209	4.8377061	27.0030134	28.4033157	153.6376268	77.1408753	55.4852971	102.9170449	69.9879256	14.242093	41.9122774	10.6142513	43.8425489	66.3166633	56.2787368	29.407552
**L-Leucine**	4.52495	6.824382	6.156799	11.008135	8.319774	13.602243	11.834081	11.624665	25.364987	11.129851	5.706917	6.225185	1.994717	1.72032	3.896821	7.780639
**L-Malic acid**	3305.154	2809.535	3741.102	6820.323	9062.78	4990.382	18301.96	8777.504	10621.715	9159.475	17843.477	3915.647	4401.045	6865.016	8258.168	15065.33
**L-Methionine**	353.4714	625.345	571.397	355.4316	398.6732	534.2654	783.1634	590.7288	559.4726	375.4622	400.9237	380.6034	811.7447	291.7093	267.0175	595.7091
**L-Phenylalanine**	1987.7731	1855.4351	1720.271	1899.2331	1014.9697	1370.1699	2522.8986	1192.4681	3113.4534	1534.3293	1550.3616	1244.1143	871.4739	776.5114	860.4978	1899.8055
**L-Proline**	0.55874233	0.62169596	1.05549224	0.52737801	0.08745248	0.66807427	0.90468385	0.78697221	0.08833243	0.43351969	0.20282626	0.11300398	0.17055412	0.44820646	0.45042962	1
**L-Serine**	5.089031	5.345452	5.384461	4.565646	11.171068	13.592625	22.805991	8.440938	11.104966	6.009345	8.008544	4.184785	6.899864	5.184536	4.419478	19.696148
**L-Sorbose**	1426.7699	1072.9037	1087.4146	1006.7919	902.0586	951.7217	1624.1049	993.7725	1787.401	1492.0967	1170.107	783.6796	404.0634	560.0692	679.1959	994.5562
**L-Threonine**	20.71261	18.56308	31.48857	24.09948	85.03932	43.14318	52.32595	91.77719	42.53304	46.2567	35.48494	27.86876	43.47852	54.16292	24.24652	83.82473
**L-Tryptophan**	841.8554	1188.1342	904.3713	745.0699	911.9394	680.7176	1064.3622	814.7542	783.7534	609.0168	590.8425	536.8286	268.5579	404.8335	300.3927	516.7631
**L-Tyrosine**	262.97007	341.27448	476.78979	217.47085	166.26494	298.92893	331.70849	140.76156	425.0684	246.01759	306.89764	279.10237	92.33424	102.31422	101.98967	269.59533
**Maleic acid**	27.04244	33.36152	20.01098	43.97509	47.47526	86.61979	24.02135	49.72921	39.11078	16.36125	13.64361	26.97708	22.83527	26.20062	21.66683	34.62434
**Malonic acid**	269.73708	98.8281	104.37382	113.95791	65.38785	481.3424	468.58881	370.17312	1135.73488	315.33812	31.16816	248.00349	284.06803	44.10229	264.29879	357.15738
**Melibiose**	1.0695459	0.9076948	0.4085508	1.330185	0.5091301	0.4332587	1	0.5603684	1.1156537	1.2630998	1.3475755	0.7344421	0.2701331	0.3551935	0.4280415	0.3993748
**Mevalonic acid 5-phosphate**	0.053297055	0.022142295	0.061299522	0.054278469	0.050350616	0.05124568	0.117739611	0.081006736	0.1472897	0.022389527	0.181459938	0.052725629	0.018382928	0.080681066	0.05785541	0.008688076
**Mevalonic acid**	0.04740405	0.1445212	0.30712892	0.17922766	0.22172798	0.15454695	0.590394	0.12331506	0.23270662	0.11339968	0.14116989	0.07843449	0.58561711	0.19490668	0.0711989	0.64422053
**m-Hydroxybenzoic acid**	8.41112186	4.04814077	8.40797204	6.1471588	1.43821312	1.4557356	7.54491953	0.83258933	0.53186849	0.07465386	5.11900454	2.43115227	0.79574426	3.74778836	3.76481896	4.31538411
**myo-Inositol**	9074.151	6859.535	7225.012	9338.735	6439.682	6483.38	10207.7	6577.84	13228.185	9623.677	7636.489	4977.305	2664.58	3694.937	4448.745	5531.382
**N-Acetyl D-galactosamine**	0.8637472	0.8329246	0.7791345	0.4286238	2.445659	2.2426139	1.2354337	1.3292577	1.1319014	0.6911224	1.172979	0.8962106	1.3510835	1.3656413	1.4471838	1.2745467
**N-Acetyl-alpha-D-glucosamine 1-phospha**	4.7091827	2.3364424	4.5340331	1.34291	4.4769078	0.7373267	0.5636785	1.5211894	0.7027911	0.1848703	0.2563102	0.3364255	7.1894905	1.0809654	4.3106165	2.472821
**N-Acetyl-D-glucosamine 6-phosphate**	0.9575768	4.7292832	3.3966255	13.0540548	4.4760197	5.0076491	3.4256855	4.6423149	2.7702379	2.209263	3.2892275	4.8414853	2.0558921	5.6330977	1.8897181	1.1767159
**N-Acetylglutamic acid**	24.81345	19.17779	32.7421	41.88609	23.46632	27.70088	51.01476	23.03101	72.91214	68.17848	89.79843	23.21103	29.75238	23.41275	21.46455	120.56902
**N-Acetylneuraminic acid**	0.5688716	3.6096301	0.3136812	10.5475123	49.987389	22.2678778	48.855067	25.3980769	0.7251187	10.1191448	16.5335734	5.3853147	29.1446644	38.0449439	40.3539564	84.0161622
**N-Carbamoyl-DL-aspartic acid**	15.62196	20.91765	26.50162	20.54054	68.50923	17.48062	36.14929	35.89861	19.55658	17.5287	28.91413	15.62105	32.12177	22.45256	18.01892	37.69737
**N-Carbamyl-L-glutamic acid**	0.11679247	0.53601101	0.09127275	2.27386778	0.11772385	0.80604171	0.15854077	0.15365662	1	0.09423388	0.3863166	0.98012256	1.10597358	0.81244475	0.92062835	0.92870456
**N-Formyl-L-Tyrosine**	0.18958518	0.02891204	0.369598797	0.068012151	0.050170808	0.064341546	0.133823387	0.096026206	0.085538238	0.119862839	0.195033283	0.139768376	0.013013917	0.01773384	0.030254779	0.009357599
**Nicotinic acid mononucleotide**	0.08497289	0.035271843	0.271448604	0.102748011	0.055009019	0.142562007	0.173367807	0.033323834	0.072184437	0.037464746	0.060264646	0.03231128	0.007402291	0.154868346	0.056920602	0.026127081
**Nicotinic acid**	1.0335079	3.8438821	4.9935813	10.4176574	5.094366	1	3.6565378	5.6030275	5.0758574	4.3381348	5.7516941	0.4332643	1.3798624	4.0877237	1.730571	3.755807
**o-Hydroxy hippuric acid**	1.1655676	3.8415739	0.5891614	1.5817742	1.1487224	1.4200547	37.786477	25.3007814	35.6863352	0.2120636	19.6147418	2.3336656	15.2957518	18.2607573	17.8721414	19.4287761
**o-Phospho-L-Serine**	0.03784026	0.02928093	0.07901628	0.04545829	0.12263689	0.04820981	0.02535741	0.07511063	0.04222775	0.02977601	0.0269324	0.07075549	0.03705016	0.03062333	0.02944037	0.04017277
**O-Phosphorylethanolamine**	0.02463904	0.059924434	0.084228998	0.03389025	0.083616671	0.159888433	0.145158063	0.231239821	0.081366572	0.040916592	0.037099196	0.026745869	0.019608182	0.008779951	0.027688725	0.02211756

**Orotic acid**	10.6353778	28.2228823	26.0009066	11.4580975	65.6114936	12.8707679	20.3633996	29.6142525	35.5263333	0.7810596	11.8872492	13.8545222	13.9348746	15.5740418	11.8097276	21.8060423
**O-Succinyl-L-homoserine**	0.72650466	9.29822502	0.33186934	1.51483231	1.78582053	1.25886354	0.07410025	0.87607454	2.5320654	6.39304035	2.33862174	0.96489116	4.6142085	1.79118841	1.17381181	0.18822279
**Oxamic acid**	0.263739281	0.019388248	0.102343217	0.021629235	0.127028632	0.070912255	0.014196147	0.048795168	0.35723472	0.12507275	3.795140181	0.045419741	0.18183078	0.076826558	0.045923984	0.007348051
**Phenylpyruvic acid**	85.53338	37.67909	68.29974	74.12495	16.26408	31.69179	64.87049	33.28819	297.19949	61.83541	120.98398	43.5151	11.65828	26.68131	22.62059	56.9879
**Phosphoenolpyruvic acid**	1.6303856	1.2381117	0.2964832	2.370477	5.7914277	0.7591647	10.2619166	1.2319255	0.308313	0.6251913	0.5571945	0.9208046	3.3803241	10.9433398	0.3716569	1.6592452
**Prephenic acid**	0.68927645	0.31479773	0.447855	0.90008314	0.20831101	0.7070545	1.02358855	0.504322	1.33883388	0.75553035	0.41190262	0.30987006	0.08056539	0.60823444	0.17655564	0.02901105
**Pyridoxal 5 phosphate**	0.075336759	0.017127015	0.01110873	0.014943375	0.050115883	0.022589205	0.046626203	0.055704192	0.021255068	0.018719933	0.089694486	0.035136016	0.006347538	0.029838638	0.013700744	0.026967082
**Pyridoxal hydrochloride**	42.971801	22.932938	33.784443	47.40208	17.408739	14.244428	24.452615	41.440032	33.862064	20.42786	31.767489	20.983786	8.180867	8.159778	7.673163	12.723306
**Pyridoxamine**	0.11679365	0.04288198	0.01456488	0.0218282	0.03771099	0.04293612	0.1148956	0.02387835	0.14657243	0.08577451	0.04930666	0.01536083	0.01852693	0.06617169	0.02127728	0.01064408
**Pyridoxine**	0.044493597	0.007196009	0.006295666	0.023729212	0.070349787	0.019431254	0.042401331	0.036549522	0.056198589	0.025989017	0.004748002	0.023223903	0.033031548	0.006200268	0.027843976	0.018959462
**Pyruvic acid**	35.02923	27.875	19.36074	18.29951	40.46649	23.4249	80.03653	41.41164	36.17542	10.89089	34.39794	15.39854	14.66961	22.15831	26.99408	62.41118
**Quinic acid**	0.13778051	0.18000788	37.16565924	0.38161654	0.97052181	0.41578876	0.54156989	28.33782229	0.32763342	0.1548903	0.06937234	1.49746128	0.24567657	0.10987652	2.19237885	0.49173379
**Riboflavin**	4.515492	2.785324	3.313833	4.71573	12.306436	6.155485	11.413775	9.051999	6.661967	4.589127	5.671828	2.663282	4.73074	5.082106	4.419727	9.686734
**Ribonic acid gamma lactone**	0.20691163	0.23131798	0.2348918	0.13086506	0.15514418	0.30344788	0.42818377	1.43016818	0.13928131	1	0.12720405	0.2009589	0.03274399	0.41875554	0.07986553	0.1099699
**S-2-Aminoethyl-L-cysteine**	0.002901588	0.00923276	0.022540821	0.003613196	0.00577006	0.027189538	0.010029739	0.073024269	0.005042682	0.067568963	0.006095564	0.002396469	0.001504584	0.019494938	0.003383335	0.0021842
**S-5-Adenosyl-L-homocysteine**	0.19894066	2.0686985	0.02615313	0.07931886	0.54488491	0.05262932	0.06323973	0.69695247	0.05439666	0.21629759	0.20392384	0.04033733	0.0729446	0.2750385	0.01637844	0.04953528
**Salicylic acid**	329.8524	310.4768	483.8678	766.9512	833.9231	466.9752	1069.2726	1029.118	1287.4672	227.151	415.0164	281.2373	597.7789	961.1854	621.6994	927.7229
**Shikimic acid**	0.03113439	0.08934497	0.21284233	0.07301461	0.04906816	0.01708142	0.02615886	0.08620594	0.07827334	0.02780046	0.06830827	0.077785	0.01240901	0.06609093	0.02850468	0.06150173
**Succinic acid**	1179.095	1238.372	1379.813	1512.976	3196.488	2037.588	4982.965	2879.364	6130.856	2281.244	3492.275	1565.839	1876.563	1815.952	2101.673	6417.874
**Succinic semialdehyde**	0.3438837	0.30598756	0.27655161	0.18199817	0.14300014	0.70279983	0.17117968	0.53032019	0.59531827	0.73101034	0.04037461	0.34842622	0.01776637	0.13568803	0.04517473	0.0960843
**Taurine**	194.43822	84.90006	183.90929	199.56113	326.55769	190.33069	248.28945	361.59651	204.97762	287.77536	195.7684	93.20726	256.43602	217.48428	195.67688	377.40196
**Taurocholic acid**	20.01509	52.74035	77.07931	63.96632	44.03781	140.4937	192.39049	67.90539	204.77907	173.61252	249.18632	97.38499	156.94134	82.85692	261.79531	53.25747
**Thiamine**	1.12720448	0.09299369	0.08005223	0.08784045	1.16479442	0.28651631	0.88074909	0.34875937	0.34123027	0.11856902	0.28938846	0.13480289	0.12310614	0.3848235	0.31782153	0.22388268
**Thymidine 5-diphosphate**	0.077730288	0.007209742	0.061177445	0.035654943	0.070996636	0.156383312	0.123939315	0.075204973	0.122574033	0.100632316	0.175013395	0.022047304	0.013167042	0.073332296	0.01335423	0.153045699
**Thymidine**	25.4837	38.93717	20.63798	25.54321	45.89581	53.25092	53.36397	25.12125	68.50346	24.58422	20.19781	17.7291	11.75378	15.38223	12.82308	22.64527
**Thymine**	0.33192706	0.69114871	0.65941921	0.09935941	4.07702254	3.64862268	4.53930121	3.18406507	2.95812046	1.99437058	1.99590302	1.16350323	1.44532693	2.12169763	1.00078347	0.97376395
**trans-4-Hydroxy-L-proline**	0.06514946	0.07009803	0.09037278	0.04188319	0.23771131	0.25884133	0.34638653	0.22433999	0.07759717	0.0626705	0.04229308	0.0326186	0.07351541	0.03848751	0.13488306	0.1068863
**trans-Aconitic acid**	523.1912	682.5211	1453.1724	1005.5091	2891.6146	776.654	2318.7474	755.0236	4266.8872	1597.0428	1201.7106	880.8064	1747.1306	856.9428	930.1368	1082.3195
**trans-trans Muconic acid**	0.024343916	0.027215752	0.060941276	0.030727941	0.023928524	0.032240114	0.008382271	0.002059461	0.013340972	0.047688435	0.021664256	0.016039198	0.012392712	0.009409097	0.013335116	0.043476736
**Trehalose 6-phosphate**	0.10926377	0.12882636	0.0532332	0.0881463	0.02758386	0.07279972	0.08814052	0.07584365	0.01923868	0.01938302	0.07759289	0.04245136	0.03412834	0.01730092	0.09963334	0.13674588
**Trehalose**	0.8553901	0.5101067	0.780271	0.635022	0.5168707	0.7146772	0.6576389	0.2199471	1.0660047	1.2227082	0.551616	0.4046044	0.1924801	0.192104	0.3114386	0.2722653
**Uracil**	33.063274	5.174642	24.49343	1.261119	81.806346	100.812019	250.408164	78.382217	55.722572	56.288331	36.14182	21.825906	33.832382	43.90002	59.739791	158.417795
**Uric acid**	6321.8697	4582.5309	6103.8525	877.2656	5423.8641	672.6707	3254.5835	1910.9265	139.2098	1087.6152	1032.8977	5035.8736	3109.9306	1805.2304	994.5981	2357.2436
**Uridine 5′-diphosphogalactose**	17.6581199	14.1906053	15.1433512	15.8676524	7.2073714	13.6960212	5.0097214	0.7709553	23.123437	6.085056	0.6316033	6.6292227	8.4997606	31.2383713	2.23123	5.0486764
**Uridine 5-diphosphate**	0.05973995	0.073929444	0.01076934	0.184765898	0.02649034	0.053931604	0.013997341	0.011158899	0.059256201	0.05705908	0.013859659	0.007663177	0.021509888	0.042050608	0.00811383	0.00962856
**Uridine 5-diphosphoglucose**	17.6581199	14.1906053	15.1433512	15.8676524	7.2073714	13.6960212	5.0097214	0.7709553	23.123437	6.085056	0.6316033	6.6292227	8.4997606	31.2383713	2.23123	5.0486764
**Uridine 5-monophosphate**	5.71430312	0.7034941	0.97258615	2.3946915	1.01928498	0.80388592	0.02400367	0.58089886	0.7510917	0.22853267	0.65631528	2.45276198	1.65497609	3.89671466	1.21326686	0.19110169
**Uridine 5-triphosphate**	0.049077733	0.019134367	0.051241206	0.050446256	0.016805628	0.014000282	0.036116294	0.03321787	0.021153138	0.021533729	0.034296311	0.016052333	0.003480788	0.015033264	0.015975416	0.04418328
**Uridine**	324.9264	69.36707	243.38638	155.19493	110.40218	295.16479	236.04111	105.18128	222.09031	84.45513	91.07905	179.25362	46.84741	98.85334	83.22474	216.60373
**Vanillic acid**	1.2727152	0.4931905	0.5794625	0.2502396	0.7629475	0.2198843	0.7832157	0.4071818	2.1388803	0.8314462	0.3888596	0.2185147	0.9793995	0.5842783	0.1573207	1.6390328
**Xanthine**	7.37E-01	6.98E-02	3.51E+02	2.34E-02	6.91E-03	1.10E-01	1.48E-01	5.10E-02	7.41E-02	1.95E-01	1.71E-02	6.59E+01	6.76E+02	4.32E-02	3.15E-02	2.70E-01
**Xanthosine**	249.04545	234.74997	270.50046	76.41716	508.85908	30.62075	91.51357	76.70194	153.38873	26.41666	32.13758	485.46687	715.72642	34.56154	33.80501	38.80797
**Xylitol**	1.0666891	0.656876	0.4677887	0.6477543	2.1958953	1.277244	0.9083101	0.4905191	2.0126279	1.3320049	1.4376948	0.9111761	1.5742927	0.7420066	0.7647662	1.139168

Table S2: HPLC setting for Reverse Phase Chromatography

HPLC settings for reverse phase chromatographic separation of plasma metabolites by gradient elution, confirmatory quantification by isotope dilutional MS. Buffer A comprised of 20 mM ammonium formate and 0.05% formic acid in HPLC grade water; Buffer B comprised of acetonitrile with 0.05% formic acid, flow rate 0.6 ml/min.

Time (min)	% Buffer B
0 – 7.0	3% – 50%
7.0 – 7.4	50% - 100%
7.5 – 7.9	100%
8.0 – 10.0	3%

Table S3. MRM Mass Spectrometer Settings, confirmatory quantification by isotope dilutional MS

Positive ion mode				
Metabolite	Parent ion m/z	Daughter ion m/z	Collision energy (V)	Retention Time (min)
Tryptophan	205.2	146	25	4.98
Tryptophan-d5	210	150	25	4.94
Kynurenine	209.2	94	22	4.42
Kynurenine-d4	213.2	98	22	4.39
kynurenic acid	190.2	144	25	5.18
Nicotinamide	123.1	80	25	4.57
nicotinamide-13C6	129.1	85	25	4.59
nicotinamide mononucleotide	335.2	123	20	2.84
Nicotinamide riboside	255.0	123.0	20	3.16
Indole-3-acetic acid	176.1	130.0	25	7.28
Indole-3-propionic acid	190.1	130.0	25	8.18
NAD+	664.4	428.0	25	3.94
NAD+-d5	669.4	428.0	25	3.94
NADH	665.4	524	25	3.30
Curtain gas: 25, GS1 0, GS2 0, Spray voltage 3000V, Source temperature 400˚C.				
Declustering potential 75, Entrance potential 5, collision excitation potential 15, collision gas 6.				
Negative ion mode				
Quinolinic acid	166.1	78	-25	2.91
Quinolinic acid-13C3, 15N1	170.1	82	-25	3.03
Indoxyl sulfate	212.2	80	-25	554
Curtain gas: 25, GS1 0, GS2 0, Spray voltage -3000V, Source temperature 400˚C.				
Declustering potential -75, Entrance potential -5, collision excitation potential -15, collision gas Medium.				

Supplemental Figure 1. Phenotypes of Sham and Uninephrectomized mice during Pregnancy

(A) Correlation of plasma cystatin C levels with directly measured GFR measured as described in Methods (n=17) in mouse models with varying degrees of renal insufficiency. Plasma cystatin C levels showed a strong negative correlation with GFR using both linear (R^2= 0.82) and polynomial models (R^2= 0.82). **(B)** Correlation of plasma creatinine levels with glomerular filtration rate (GFR) measured as described in Methods (N=17). Plasma creatinine level showed a modest relationship with measured GFR using both linear (R^2= 0.53) or power models (R^2= 0.71) **(C)** Plasma cystatin C measurements at baseline, Day 1, Day 7 and Day 14 after surgery in control (Sham) and uninephrectomized (UNx) mice. **(D)** Estimated glomerular filtration rate (eGFR) at baseline, Day 1, Day 7 and Day 14 after surgery was calculated in Sham and UNx mice as described in Methods. **(E)** Body weights of Sham and UNx mice throughout pregnancy. **(F)** Gestational day 18 (GD18) left kidney weights in Sham and UNx mice. Data were normalized with body weight values. Data depicted as mean ± standard deviation. n=6 per group except otherwise indicated. Unpaired 2-tailed *t* test; $^*P < 0.05$ in UNx versus Sham mice.

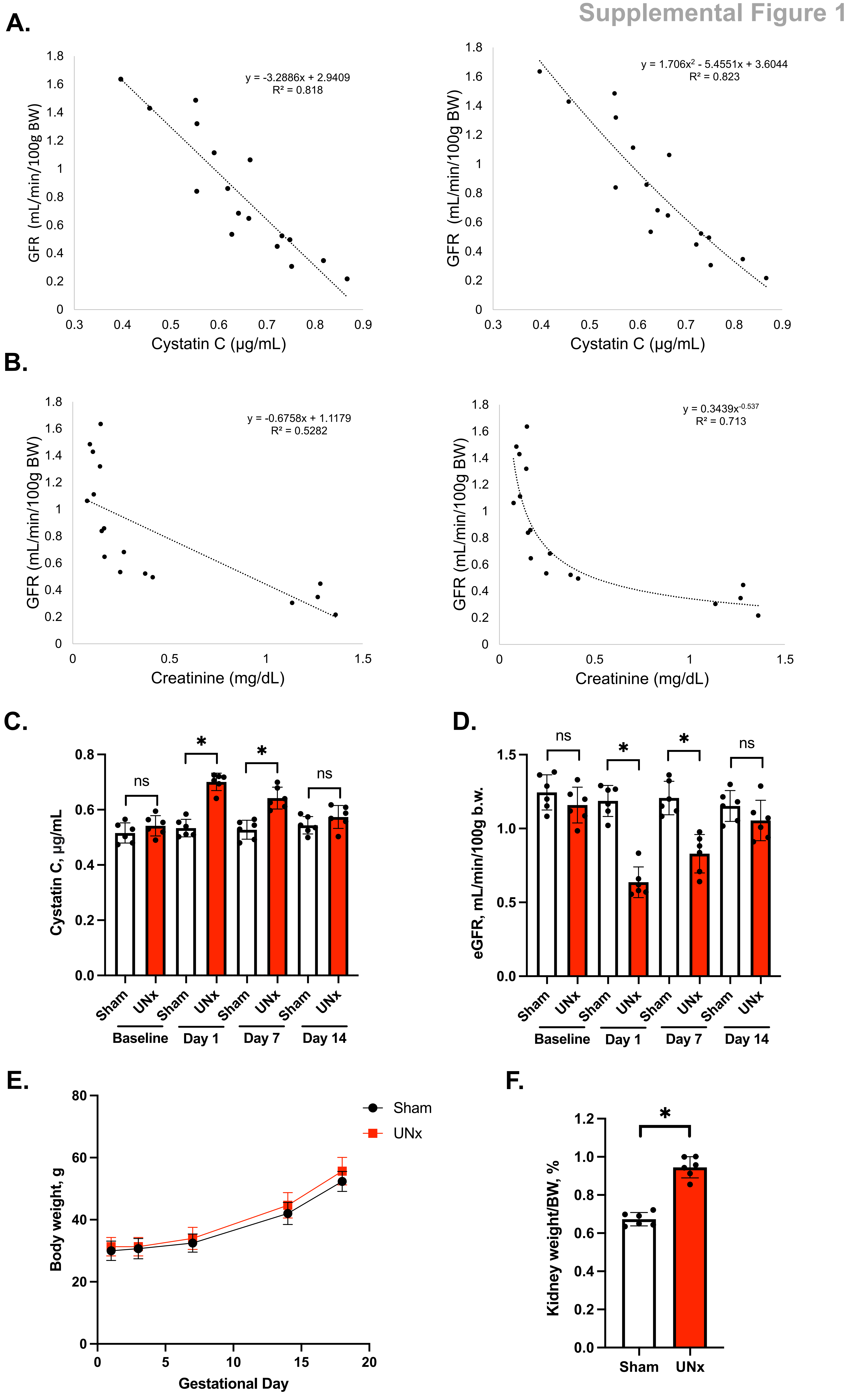
A.
y = -3.2886x + 2.9409
R² = 0.818
GFR (mL/min/100g BW)
Cystatin C (µg/mL)
y = 1.706x² - 5.4551x + 3.6044
R² = 0.823
GFR (mL/min/100g BW)
Cystatin C (µg/mL)
B.
y = -0.6758x + 1.1179
R² = 0.5282
GFR (mL/min/100g BW)
Creatinine (mg/dL)
y = 0.3439x-0.537
R² = 0.713
GFR (mL/min/100g BW)
Creatinine (mg/dL)
C.
Cystatin C, µg/mL
ns
*
*
ns
Sham
UNx
Baseline
Day 1
Day 7
Day 14
D.
eGFR, mL/min/100g b.w.
ns
*
*
ns
Sham
UNx
Baseline
Day 1
Day 7
Day 14
E.
Body weight, g
Gestational Day
Sham
UNx
F.
Kidney weight/BW, %
*
Sham
UNx

Supplemental Figure 2. Additional Features of the Impact of Prior Uninephrectomy on Pregnancy in mice

(A) CD34 immunohistochemistry analysis of renal tissue at GD18 from one representative control (Sham, left panel) and one uninephrectomized (UNx, right panel) mice. UNx mice exhibit loss of CD34 positive endothelial cells among glomerular capillaries (scale bar= 125μm). Summary data for the CD34 positive glomerular area quantified using ImageJ software in Sham and UNx mice at GD18 is depicted. GD18 plasma aspartate aminotransferase (AST) **(B)** and alanine aminotransferase **(C)** measurements in Sham and UNx mice. **(D)** Plasma volume measurements in Sham and UNx mice in non-pregnant (NP) state, at GD14 and GD18, and shown as change when compared with NP values **(E)**. Data were normalized with body weight values. Data depicted as mean ± standard deviation. n=6 per group except otherwise indicated. Unpaired 2-tailed *t* test; *$P < 0.05$ in UNx versus Sham mice.

Supplemental Figure 2

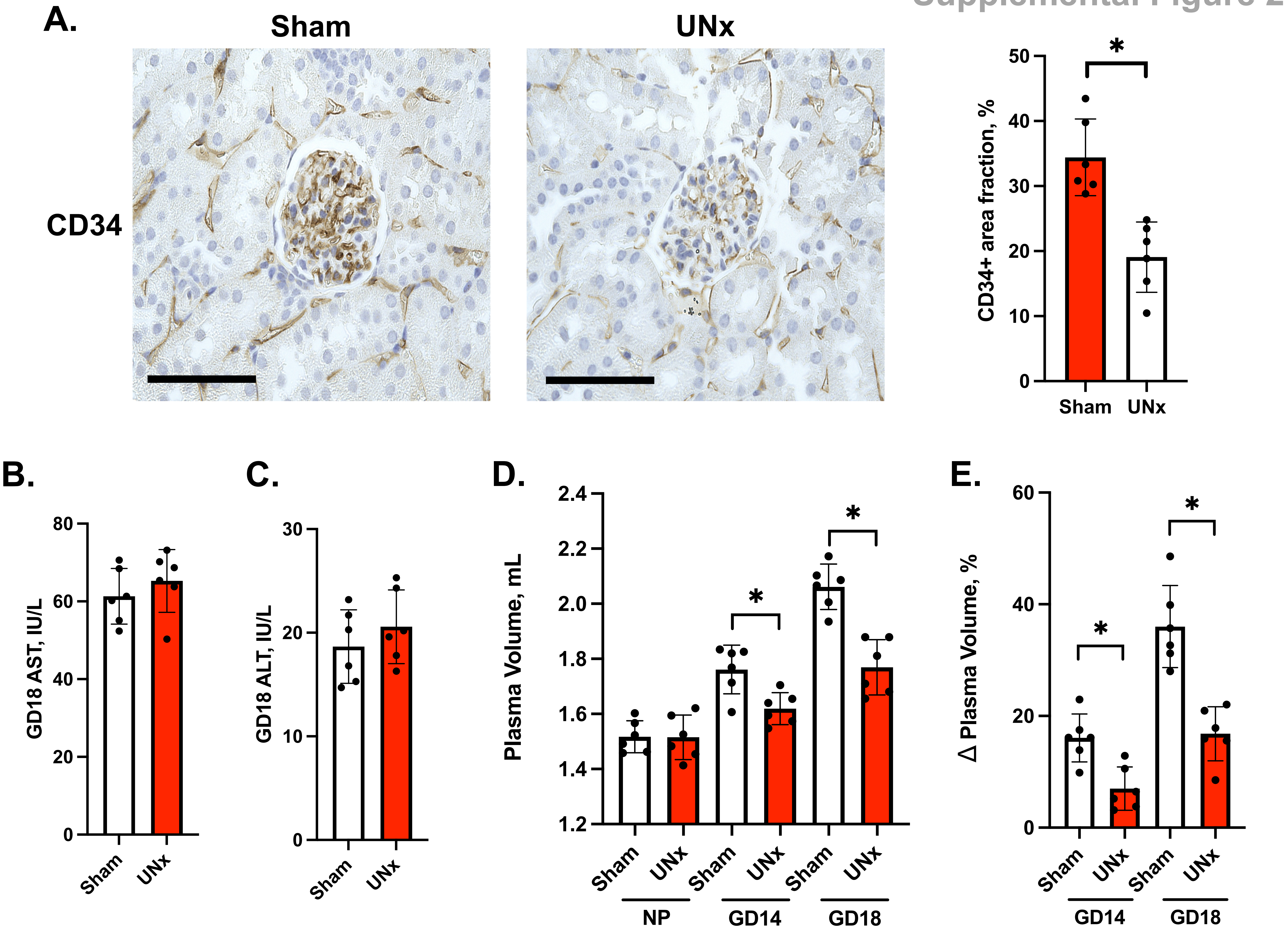

Supplemental Figure 3. Phenotypes of Uninephrectomized mice treated with PBS or Placental Growth Factor

(A) Body weights of uninephrectomized (UNx) mice treated with either PBS or placental growth factor (PlGF) throughout pregnancy. **(B)** Gestational day 18 (GD18) left kidney weights in UNx mice treated with PBS or PlGF. Data were normalized with body weight values. **(C)** CD34 immunohistochemistry analysis of renal tissue at GD18 from one representative UNx mice treated with PBS (left panel) or PlGF (right panel). PlGF treatment increased CD34 positive area among glomeruli in late pregnant UNx mice (scale bar= 125μm). **(D)** Summary data for the CD34 positive glomerular area quantified using ImageJ software in UNx mice treated with PBS or PlGF at GD18 is depicted. **(E)** Histopathological analysis of placenta tissue from one representative UNx mice treated with PBS (upper panel) or PlGF (lower panel) at GD18 (scale bar= 125 μm). H&E stain and CD34 stain shows similar labyrinthine vasculature in UNx mice treated with PBS or PlGF. **(F)** Summary data for the CD34 immunohistochemistry quantitated within the labyrinth (using ImageJ software) between UNx mice treated with PBS or PlGF. Data depicted as mean ± standard deviation. n=6 per group. Unpaired 2-tailed *t* test; *P < 0.05 in in UNx mice treated with PlGF versus PBS.

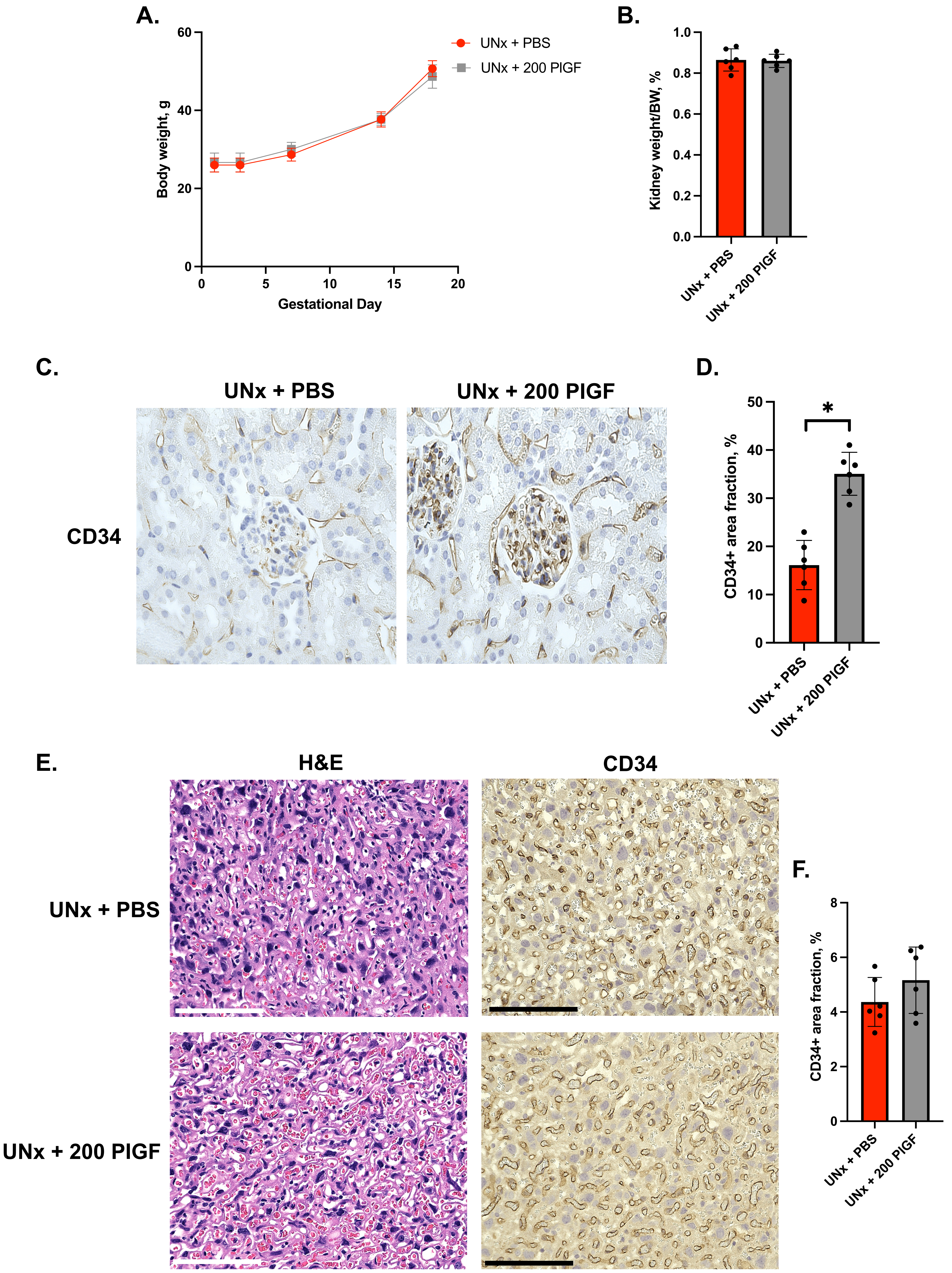

Supplemental Figure 3

Supplemental Figure 4. Trends in Metabolites Levels during Pregnancy in Sham and Uninephrectomized Mice from Metabolite platform

Levels of O-phosphorylethanolamine **(A)**, L-homocysteine **(B)**, L-carnitine **(C)**, Myo-inositol **(D)**, Melibiose **(E)** and 5-Hydroxyindoleacetic acid **(F)** on the metabolite platform in non-pregnant (NP) state and at gestational day 18 (GD18) in Sham and uninephrectomized (UNx) mice. Results shown are normalized peak intensities (arbitrary unit) as individual values and as mean ± standard deviations for each of the metabolites. n=4 per group. 1-way ANOVA with Tukey's test for multiple comparisons; *$P < 0.05$.

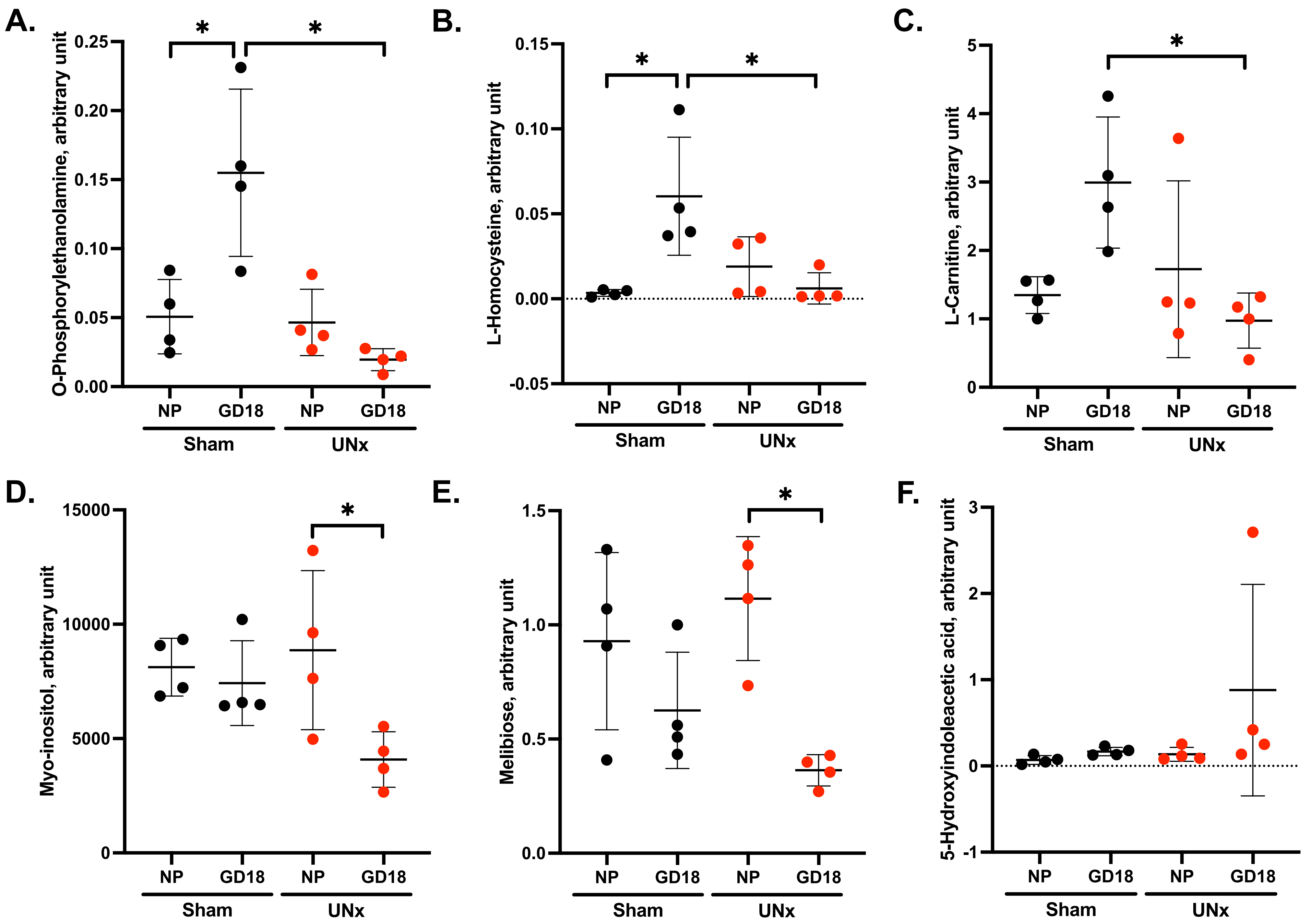

Supplemental Figure 4

Supplemental Figure 5. Phenotypes of Uninephrectomized mice treated with L-Kynurenine versus Uninephrectomized Mice during Pregnancy

(A) Body weights of uninephrectomized (UNx) mice and UNx mice treated with L-kynurenine 25mg/L added to drinking water (UNx+Kyn) throughout pregnancy. **(B)** Gestational day 18 (GD18) left kidney weights in UNx and UNx+Kyn mice. Data were normalized with body weight values. **(C)** CD34 immunohistochemistry analysis of renal tissue at GD18 from one representative UNx (left panel) and one UNx+Kyn mice (right panel). L-kynurenine supplementation increased CD34 positive area among glomeruli in late pregnant UNx mice (scale bar= 125μm). **(D)** Summary data for the CD34 positive glomerular area quantified using ImageJ software in UNx and UNx+Kyn mice at GD18 is depicted. Data represent the mean ± standard deviation. n=6 per group. Unpaired 2-tailed *t* test; **P* < 0.05 in UNx+Kyn versus UNx mice.

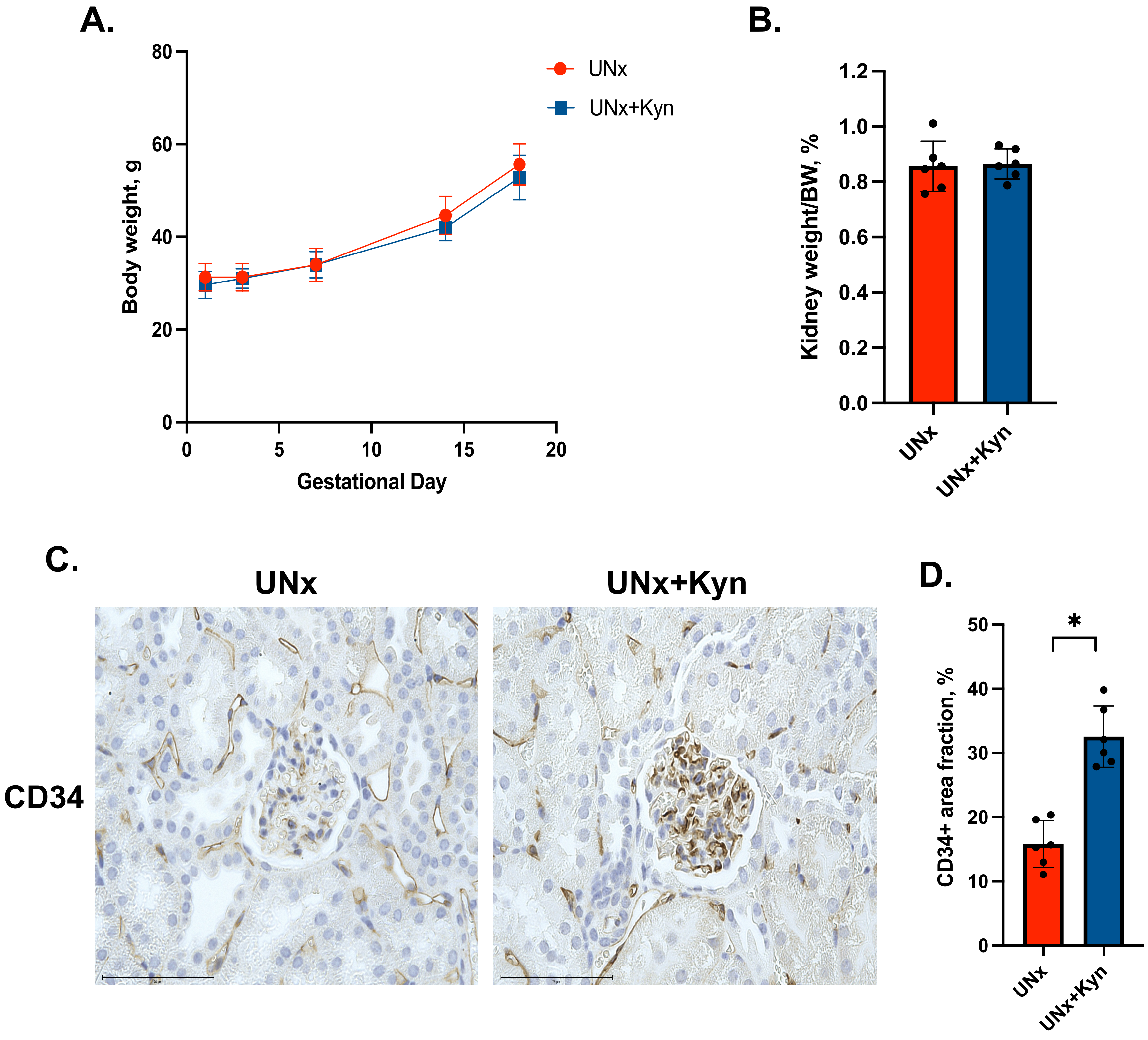

Supplemental Figure 5
